# Supplementary material for: Universal non-Hermitian skin effect in two and higher dimensions
Source: Nat Commun. 2022 May 6;13:2496. doi: 10.1038/s41467-022-30161-6 (PMC9076925; doi:10.1038/s41467-022-30161-6)
Supplement: Supplementary file 1 — Supplementary Information [file 41467_2022_30161_MOESM1_ESM.pdf]

Supplementary Information: Universal non-Hermitian Skin Effect in Two and Higher Dimensions

Kai Zhang,[1,2] Zhesen Yang,[3,*] and Chen Fang[1,3,4,†]

[1] Beijing National Laboratory for Condensed Matter Physics and Institute of Physics, Chinese Academy of Sciences, Beijing 100190, China

[2] University of Chinese Academy of Sciences, Beijing 100049, China

[3] Kavli Institute for Theoretical Sciences, Chinese Academy of Sciences, Beijing 100190, China

[4] Songshan Lake Materials Laboratory, Dongguan, Guangdong 523808, China

SUPPLEMENTARY NOTE 1: THE PROOF OF THE THEOREM

In this section, we will prove the following theorem stated in the main text:

> **Theorem**: In the thermodynamic limit, the skin effect is present in a Hamiltonian having open boundary of generic geometry, if the spectral area is nonzero; vice versa, the skin effect is absent for all possible geometries, if the spectral area is zero.

Here the spectral area refers to the area of the region covered by the periodic boundary spectrum on the complex plane. In the following contents, we will first show some numerical verifications of the theorem, and then prove the theorem in two dimensions, and finally extend the proof to three-dimensional cases.

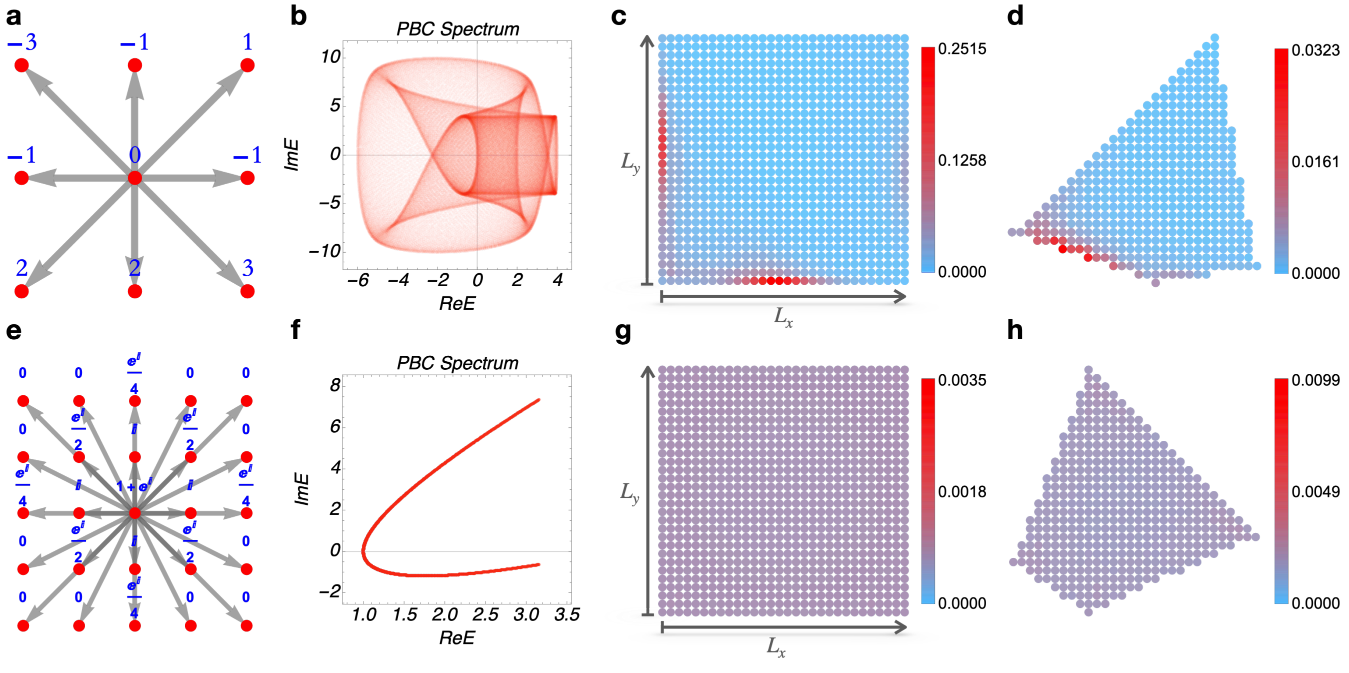

Supplementary Figure 1. Some numerical examples of the theorem. (a-d) show an example having skin effect, and (e-h) show an example without skin effect. (a) and (e) show the corresponding hopping parameters of the Hamiltonian shown in Eq. (1). (b) and (f) show the periodic boundary spectrum $E(\mathbf{k})$, where 200×200 $\mathbf{k}$-grid is used. The spatial distribution of eigenstates, that is $W(\mathbf{x})$ in Eq. (2), of these two examples on the square geometry are shown in (c) and (g), respectively, and the system size is taken as $L_x = L_y = 30$. The spatial distribution $W(\mathbf{x})$ under some generic open-boundary geometries are plotted in (d) and (h).

* yangzs@ucas.ac.cn

† cfang@iphy.ac.cn

A. Some numerical examples of the theorem

In this subsection, we provide some numerical examples of the theorem. For simplicity, we consider the following single-band model

$$\mathcal{H}(\mathbf{k}) = \sum_{i,j} t_{ij}\beta_x^{-i}\beta_y^{-j}, \qquad \beta_{x/y} = e^{ik_{x/y}}, \tag{1}$$

where t_{ij} represents the hopping parameter between the site (m,n) and the site $(m,n)+(i,j)$.

The two rows in Fig. 1 represent two different models. In the first one, the hopping parameters are shown in Fig. 1 (a), and the periodic boundary spectrum, i.e. $E(\mathbf{k}) = \mathcal{H}(\mathbf{k})$ with $\mathbf{k} \in$ BZ, is shown in Fig. 1 (b). Here the $\mathbf{k}$ 200×200 is used. One can notice that the spectral area of the first model is nonzero. As a result, the open boundary eigenstates show the localization behaviors, namely, the emergence of skin effect, as shown in Fig. 1 (c-d). In order to illustrate the localization properties,

$$W(\mathbf{x}) = \frac{1}{N}\sum_n |\psi_n(\mathbf{x})|^2 \tag{2}$$

is plotted in Fig. 1 (c-d), , where $\psi_n(\mathbf{x})$ is the n-th normalized eigenstate of the Hamiltonian with open-boundary condition, and N is the number of eigenstates. For the second example, since the spectral area is zero, as shown in Fig. 1 (f), there is no skin effect. Indeed, $W(\mathbf{x})$ shown in Fig. 1 (g-h) are uniform distributed on the lattice.

The results of the numerical calculation support our theorem, and then we will formally prove the theorem.

B. The proof of the theorem in two-dimensions

In this subsection, we will prove the theorem in two-dimensional systems. As illustrated in Fig. 2(a), the proof is divided into three steps. Therefore, we partition this subsection into three parts, and each part corresponds to one step of the proof.

1. *Step I : spectral area and spectral winding*

We begin with a two-dimensional single-band non-Hermitian model with periodic boundary in both x and y directions

$$\mathcal{H}(\mathbf{k}) = u(\mathbf{k}) + iv(\mathbf{k}), \tag{3}$$

where u and v are real functions about $\mathbf{k} = (k_x, k_y)$. For any $\mathbf{k}_r \in$ BZ, one can define the following winding number

$$\nu(\mathbf{k}_r) = \oint_{\Gamma_{\mathbf{k}_r}} \frac{d\mathbf{k}}{2\pi i}\cdot \nabla_{\mathbf{k}} \ln\det[\mathcal{H}(\mathbf{k}) - E_r], \quad \mathbf{k}_r \in \text{BZ}, \tag{4}$$

where $\Gamma_{\mathbf{k}_r}$ represents the infinitesimal counterclockwise loop enclosing $\mathbf{k}_r$. Here $E_r = \mathcal{H}(\mathbf{k}_r)$ represents the reference energy, which is shown in Fig. 2 (b) with red point. We note that for different $\mathbf{k}_r$, the reference energy is different. This topological invariant describes the spectral winding on the complex plane. As shown in Fig. 2 (b), if $\nu(\mathbf{k}_r)$ is nonzero, the image of $\Gamma_{\mathbf{k}_r}$, i.e. $\mathcal{H}(\Gamma_{\mathbf{k}_r})$, forms a closed loop that encloses E_r.

First we prove the following two statements,

1. if there are some generic $\mathbf{k}_r$ points in the BZ with nonzero topological charge, the spectral area must be nonzero;

2. if all the $\mathbf{k}_r$ points in the BZ have zero topological charge, the spectral area must be zero.

The above two statements establishes the equivalence relation between "spectral area" and "topological charge of $\mathbf{k}$ point".

Based on the definition of winding number, the statement 1 is obvious. Therefore, we only need to prove the statement 2. In order to show this, we expand the Hamiltonian at the point $\mathbf{k}_r \equiv (k_x^r, k_y^r)$ as follows

$$\mathcal{H}(\mathbf{k}) - \mathcal{H}(\mathbf{k}_r) \approx \partial_x\mathcal{H}(\mathbf{k}_r)q_x + \partial_y\mathcal{H}(\mathbf{k}_r)q_y, \tag{5}$$

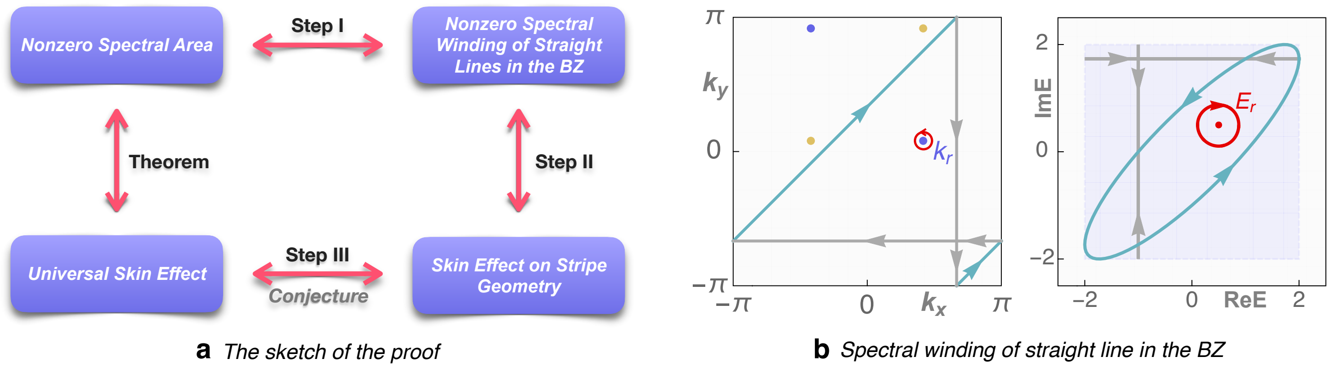

Supplementary Figure 2. (a) The outline of the proof of the theorem. (b) illustrates the spectral winding for Hamiltonian Eq. 13. Here the paths in BZ (the left-side panel) correspond to the spectral loops (or arcs) on the complex plane (the right-side panel) with the same color, respectively. The four points in BZ have the same energy E_r, and the light blue region in the right-side panel represents the periodic-boundary spectrum.

where q_x, q_y are the displacements $\mathbf{k}-\mathbf{k}_r$ in x, y directions respectively. For a generic $\mathbf{k}_r$ point, the first derivative of $\mathcal{H}$ does not vanish, i.e., the coefficients of q_x and q_y in Eq. (5) cannot be zero at the same time. The reason is that in order to make $\partial_x\mathcal{H}(\mathbf{k}_r)=\partial_y\mathcal{H}(\mathbf{k}_r)=0$, four independent real conditions, $\mathrm{Re}[\partial_x\mathcal{H}(\mathbf{k}_r)]=\mathrm{Im}[\partial_x\mathcal{H}(\mathbf{k}_r)]=\mathrm{Re}[\partial_y\mathcal{H}(\mathbf{k}_r)]=\mathrm{Im}[\partial_y\mathcal{H}(\mathbf{k}_r)]=0$, need to be satisfied. However, in two dimensions, there are only two free parameters (k_x, k_y), which cannot satisfy the above four equations generally.

In order to calculate the topological charge of a generic $\mathbf{k}_r$ point, according to Eq. (3), one can define

$$C(\mathbf{k}_r)=\begin{pmatrix}\partial_x u(\mathbf{k}_r) & \partial_y u(\mathbf{k}_r)\\ \partial_x v(\mathbf{k}_r) & \partial_y v(\mathbf{k}_r)\end{pmatrix}, \tag{6}$$

where the notation $\partial_{x/y}$ refers to $\partial/\partial_{k_{x/y}}$. When $\det[C(\mathbf{k}_r)]\neq 0$, the topological charge of $\mathbf{k}_r$ is the sign of the determinant of $C(\mathbf{k}_r)$, expressed as

$$\nu(\mathbf{k}_r)=\mathrm{sign}[\det[C(\mathbf{k}_r)]]. \tag{7}$$

Therefore, a sufficient and necessary condition for the zero charge of every $\mathbf{k}\in$ BZ (the statement 2) is

$$\det[C(\mathbf{k})]=\partial_x u(\mathbf{k})\partial_y v(\mathbf{k})-\partial_y u(\mathbf{k})\partial_x v(\mathbf{k})=0. \tag{8}$$

A theorem (the corollary of theorem 13.2) in Ref. [1] tells us that if $C(\mathbf{k})\neq 0$ and $\det[C(\mathbf{k})]=0$ for an open set S, then $u(\mathbf{k})$ and $v(\mathbf{k})$ have a functional dependent relation with $\mathbf{k}\in S\subset$ BZ, that is, $u(\mathbf{k})=g(v(\mathbf{k}))$ with g being a complex function. Applying the theorem to the entire BZ (except for some isolated points where the first derivative vanishes), one can reexpress the single-band Hamiltonian that satisfies Eq. (8) as

$$\mathcal{H}(\mathbf{k})=P[h(\mathbf{k})], \tag{9}$$

where $h(\mathbf{k})$ is a real and periodic function of $\mathbf{k}$, and P is a complex polynomial of h. Since $h(\mathbf{k})$ is a real periodic function, its image must be an arc on the real axis, e.g. $h(\mathbf{k})\in[h_1,h_2]$, where $h_{1/2}$ are real numbers. Therefore, the image of $P[h(\mathbf{k})]$ must be an arc on the complex plane, that is to say, the spectral area is zero. This completes the proof of the statement 2 for single-band models.

Generalizing the above discussion to the multi-band case, for each $\mathbf{k}_r\in$ BZ, the topological charge defined for the m-th band is

$$\begin{aligned}\nu_m(\mathbf{k}_r)&=\oint_{\Gamma_{\mathbf{k}_r}}\frac{d\mathbf{k}}{2\pi i}\cdot\nabla_{\mathbf{k}}\log\det[\mathcal{H}(\mathbf{k})-E_m(\mathbf{k}_r)]\\&=\sum_n\oint_{\Gamma_{\mathbf{k}_r}}\frac{d\mathbf{k}}{2\pi i}\cdot\nabla_{\mathbf{k}}\log[E_n(\mathbf{k})-E_m(\mathbf{k}_r)],\end{aligned} \tag{10}$$

where $E_m(\mathbf{k}_r)$ is the energy of the m-th band with the momentum $\mathbf{k}_r$. For the second equal sign in Eq. (10), we have used $\det[\mathcal{H}(\mathbf{k})-E_m(\mathbf{k}_r)]=\prod_n[E_n(\mathbf{k})-E_m(\mathbf{k}_r)]$. For Eq. (10), if $\mathbf{k}_r$ is not the degeneracy point, only $n=m$ term

in the summation has contributions to the topological charge. Therefore, the Eq. (10) further becomes

$$\nu_m(\mathbf{k}_r) = \oint_{\Gamma_{\mathbf{k}_r}} \frac{d\mathbf{k}}{2\pi i} \cdot \nabla_{\mathbf{k}} \log[E_m(\mathbf{k}) - E_m(\mathbf{k}_r)]. \tag{11}$$

Using the similar approaches in the single-band case, one can conclude that, the real and imaginary parts of $E_m(\mathbf{k})$ are locally functional dependent on the neighborhood of $\mathbf{k} \in \mathrm{BZ}$. As a result, the spectrum of $E_m(\mathbf{k})$ must be an arc. The above conclusion applies for each band.

So far, we have proven the equivalence relation between "spectral area" and "topological charge". In following contents, we will prove that if there are some generic $\mathbf{k}$ points in the BZ with nonzero topological charge, then there must be nonzero spectral winding number of the straight lines in the BZ.

We first notice that the BZ in two-dimensional systems can be covered by a set of straight lines of any slope, labeled as $\{L_s\}$. Here, the subscript s indicates the slope of the set $\{L_s\}$, and L_s represents a generic straight line belonging to $\{L_s\}$. For example, if we fix $k_y(k_x)$ and change $k_x(k_y)$ from 0 to 2π, we obtain a horizontal (vertical) straight line with the slope s being 0 (∞) in BZ, and the set of all horizontal or vertical straight lines ($\{L_0\}$ or $\{L_\infty\}$) covers the entire BZ. Particularly, an inclined straight line goes out from one side of BZ and again enters from another side as shown in Fig. 2(b). Since the straight lines on the BZ are periodic, one can define the spectral winding number for each straight lines with respect to the prescribed reference energy E_r.

$$\nu(L_s, E_r) = \oint_{L_s} \frac{d\mathbf{k}}{2\pi i} \cdot \nabla_{\mathbf{k}} \log\det[\mathcal{H}(\mathbf{k}) - E_r]. \tag{12}$$

Obviously, if all the $\mathbf{k}$ points on the BZ have zero topological charge, $\nu(L_s, E_r)$ must be zero for arbitrary L_s and E_r. Otherwise, one can always find some L_s, such that $\nu(L_s, E_r)$ is nonzero. Next we briefly prove the latter statement. Assuming that each straight line in $\{L_0\}$ and $\{L_\infty\}$ has zero spectral winding, and there is a k_r point carrying nonzero topological charge on the BZ. For a generic inclined straight line, one can always find corresponding horizontal and vertical straight lines, such that together with the inclined straight line to form a closed path enclosing $\mathbf{k}_r$ in BZ. Therefore, the closed path has nonzero winding number with respect to E_r. Due to the zero spectral winding of the horizontal and vertical straight lines as we assumed, hence a generic inclined straight line must have nonzero spectral winding number.

Let's take an example to show this. Consider a simple single-band model with the Hamiltonian

$$\mathcal{H}(k_x, k_y) = 2\cos k_x + 2i \sin k_y. \tag{13}$$

As shown in Fig. 2, the spectrum of the Hamiltonian along each horizontal or vertical straight line (gray lines) in the BZ has zero spectral winding number with respect to any reference energy. However, if we choose the straight line in the dark cyan color, these three straight lines (two gray lines and the darker cyan line) together form a closed path that encloses $\mathbf{k}_r$. Therefore, the closed path has nonzero spectral winding number with respect to E_r. Due to the zero spectral winding of two gray lines, the dark cyan straight line must have nonzero winding number regarding E_r as illustrated in Fig. 2(b).

In conclusion, we have proven the equivalence between "spectral area" and "spectral winding number of straight lines in the BZ". Up to this point, we have completed the first step of the proof shown in Fig. 2(a).

2. Step II : spectral winding and skin effect on the stripe geometry

Before, we have proved that if the spectral area is zero, the spectral winding number along each direction in the BZ is zero, and vice versa; if the spectral area is nonzero, there is at least one direction along which the spectral winding number is nonzero.

Now we transform the momentum basis from $\mathbf{k} = (k_x, k_y)^T$ to $\mathbf{q} = (q_\parallel, q_y)^T$ by

$$\mathbf{q} = S\,\mathbf{k}; \quad \det S = 1, \tag{14}$$

where S is a $2 * 2$ integer matrix due to the lattice momentum in the BZ is discrete. For any direction in the BZ, one can always choose the appropriate $\mathbf{q}$ basis such that under this $\mathbf{q}$ basis, each straight line in this direction can be obtained by fixing $q_\parallel$ and running q_y from 0 to 2π. Accordingly, the Hamiltonian $\mathcal{H}(k_x, k_y)$ can be transformed into $\tilde{\mathcal{H}}(q_\parallel, q_y)$. For any fixed $q_\parallel$, the spectral winding can be expressed as

$$w_{E_b}(q_\parallel) = \frac{1}{2\pi i} \int_{-\pi}^{\pi} dq_y \partial_{q_y} \log\det[\tilde{\mathcal{H}}(q_\parallel, q_y) - E_b], \tag{15}$$

where E_b is the reference energy.

According to what we have proved, if the spectral area is zero, under any transformation S, the spectral winding $w_{E_b}(q_{\|}) = 0$ for any $q_{\|}$ and E_b. It means that the Hamiltonian on any stripe geometry does not exhibit skin effect if spectral area is zero. Here the stripe geometry refers to the geometry with open boundary in only one direction and periodic boundary in other direction (preserves the momentum $q_{\|}$). If the spectral area is nonzero, there is at least one stripe geometry on which the Hamiltonian establishes skin effect.

Therefore, we have completed the second step of the proof.

3. Step III : the conjecture

Conjecture: For a given edge, if it does not exhibit skin effect under any types of stripe geometry, then it does not show skin effect under any fully open boundary geometry, vice versa — if it does not show skin effect under any fully open boundary geometry, then it does not exhibit skin effect under any types of stripe geometry.

Note that the above is the equivalent statement of the conjecture in step III of Fig. 2(a), that is, skin effect on stripe geometry implies skin effect on fully open-boundary geometry (i.e., the universal skin effect).

Next we explain why the conjecture makes sense, and take some numerical results to support our conjecture.

- First, we believe that whether a given edge exhibits a skin effect or not depends only on the bulk spectral topology in the direction perpendicular to this edge, having nothing to do with other boundary conditions, which accords with the spirit of bulk-boundary correspondence.
- Second, for a given edge, no skin effect means that the wave packet obeys the conventional law of reflection on this edge (as shown in Fig.4(c) of the main text). Otherwise, the skin effect leads to the anomalous dynamical behavior on this edge (as shown in Fig.4(d)). This local physical consequence caused by the skin effect can not be affected by any change to other edges in the thermodynamic limit. Therefore, we conjecture that if a given edge under stripe geometry does not exhibit the skin effect, it still does not show the skin effect under any open boundary geometry, and vice versa.

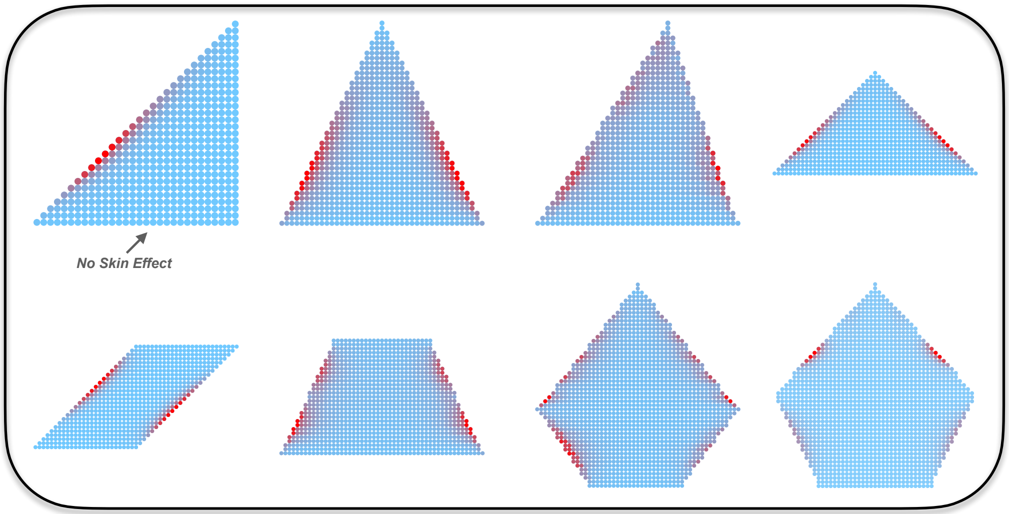

Supplementary Figure 3. Some numerical examples to support the conjecture. The lower open boundary has always no skin effect, regardless of the shape of the fully open-boundary geometry.

- Third, we show the numerical results in Fig. 3 to support the conjecture. The bulk Hamiltonian of this model reads

$$\mathcal{H}(\mathbf{k}) = 2\cos k_x + i\cos k_y. \tag{16}$$

The mirror-y symmetry of the bulk Hamiltonian makes the zero spectral winding for any a k_x-subsystem. As a result, the Hamiltonian on the stripe geometry with only open boundary in y direction does not show the skin effect. Even if opening the boundary in x direction and deforming the geometry into some generic open-boundary geometries, there is still no skin effect on the y-directional open boundary, as shown in Fig. 3.

Based on the first two steps of the proof and the above conjecture, the theorem of the universal skin effect can be obtained.

C. The proof of the theorem in three-dimensions

In this section, we extend the above proof of two-dimensional systems into three dimensions, and obtain the conclusion that nonzero spectral area means the existence of the universal skin effect.

Consider a general three-dimensional single-band tight-binding Hamiltonian, which consists of real- and imaginary-part functions

$$\mathcal{H}(k_x, k_y, k_z) = u(k_x, k_y, k_z) + iv(k_x, k_y, k_z). \tag{17}$$

We choose a generic $\mathbf{k}_r$ point and use its energy $\mathcal{H}(\mathbf{k}_r)$ as the reference energy. For a given reference energy E_r, we can obtain a one-dimensional curve in the there-dimensional BZ by solving the following two real equations,

$$\begin{aligned} u(k_x, k_y, k_z) &= \mathrm{Re}(E_r); \\ v(k_x, k_y, k_z) &= \mathrm{Im}(E_r). \end{aligned} \tag{18}$$

Each equation determines a surface, and the intersection of two surfaces is one-dimensional curve in there-dimensional BZ. The tangent direction of the curve at $\mathbf{k}_r$ is perpendicular to the normal vector of the two surfaces at this $\mathbf{k}_r$ point. The tangent vector at $\mathbf{k}_r$ is expressed as

$$\boldsymbol{T}_{\mathbf{k}_r} = \boldsymbol{\nabla} u(\mathbf{k}_r) \times \boldsymbol{\nabla} v(\mathbf{k}_r), \tag{19}$$

where $\boldsymbol{\nabla} u(\mathbf{k}_r)$ represents the gradient of u. We choose the local coordinate system (R^3 space) with $\mathbf{k}_r$ as the origin, and the gradient is reexpressed as

$$\boldsymbol{\nabla} u(\mathbf{k}_r) = \partial_x u(\mathbf{k}_r) q_x + \partial_y u(\mathbf{k}_r) q_y + \partial_z u(\mathbf{k}_r) q_z, \tag{20}$$

where $q_i \equiv (\frac{\mathbf{k}-\boldsymbol{k_0}}{|\mathbf{k}-\boldsymbol{k_0}|})_i$, $\mathbf{k}$ and $\boldsymbol{k_0}$ represent two vectors in the global coordinate system.

Next we expand the Hamiltonian into Taylor series around the origin of the local coordinate system,

$$\mathcal{H}(\mathbf{k}) - \mathcal{H}(\mathbf{k}_r) = \sum_{i=x,,y,z} \partial_i \mathcal{H}(\mathbf{k}_r) q_i + o(|\boldsymbol{q}|), \tag{21}$$

where the subscription i represents the partial differential to x, y, z. And q_i represents the deviation of k_i from $k_{r,i}$ and the last term is the infinitesimal of higher order of $|\boldsymbol{q}|$. Obvious, the zero winding condition requires

$$\begin{aligned} \partial_x u(\mathbf{k}_r)\partial_y v(\mathbf{k}_r) - \partial_x v(\mathbf{k}_r)\partial_y u(\mathbf{k}_r) &= 0; \\ \partial_x v(\mathbf{k}_r)\partial_z u(\mathbf{k}_r) - \partial_x u(\mathbf{k}_r)\partial_z v(\mathbf{k}_r) &= 0; \\ \partial_y u(\mathbf{k}_r)\partial_z v(\mathbf{k}_r) - \partial_y v(\mathbf{k}_r)\partial_z u(\mathbf{k}_r) &= 0, \end{aligned} \tag{22}$$

or equivalently,

$$\boldsymbol{T}_{k_r} = \mathbf{0}. \tag{23}$$

Next, we prove that if all $\mathbf{k}$ points in three-dimensional BZ satisfy $\boldsymbol{T}_k = \mathbf{0}$, then the entire 3D periodic-boundary spectrum must be an arc in the complex plane. We define a two-tuple function $W(\mathbf{k}) := [u(\mathbf{k}) \quad v(\mathbf{k})]^t$ with three variables, the exterior derivative of the vector-valued function is expressed as

$$dW = \begin{pmatrix} \partial_x u(\mathbf{k}) & \partial_y u(\mathbf{k}) & \partial_z u(\mathbf{k}) \\ \partial_x v(\mathbf{k}) & \partial_y v(\mathbf{k}) & \partial_z v(\mathbf{k}) \end{pmatrix}. \tag{24}$$

Eq. (23) implies that the rank of dW less than 2 (the number of components of W). To be precise, there are the following cases. (i.) Both the gradients of u and v are not zero vector, and they are linearly dependent on each other. (ii.) One of the gradients of u and v is zero vector. (iii.) Both the gradients of u and v are zero vector. In all these cases, we can obtain the final conclusion that u and v are linearly functional dependent on each other. Therefore, the spectrum must be arcs on the complex plane.

A 3D BZ can be divided into a series of plane systems, each plane corresponds to a two-dimensional subsystem. If the spectral area of a three-dimensional system is nonzero, then for each reference energy on the spectral area, its preimage (1D ring) has nonzero topological charge. Equivalently, the two-dimensional subsystem, of which the BZ (2D plane) has intersections with the ring, also has nonzero topological charge for the intersecting k points. Hence, the 2D subsystem has nonzero spectral area, and has the universal skin effect. Correspondingly, we come to the same conclusion in 3D systems that nonzero spectral area implies the presence of the universal skin effect.

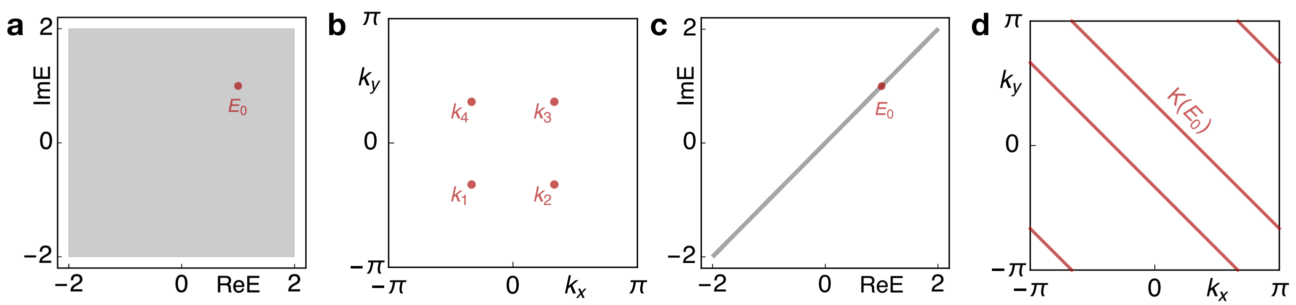

Supplementary Figure 4. (a) shows the periodic-boundary spectrum of Eq. (28) with gray color, and the pre-images of $E_0 = 1 + i$ (red point in (a)) are the four red points in (b). The periodic-boundary spectrum of Eq. (29) is the gray line in (c), and $\mathbf{k}(E_0 = 1 + i)$ is plotted by the red lines in (d).

SUPPLEMENTARY NOTE 2: A PHYSICAL EXPLANATION FOR THE THEOREM

Here, we use some examples to illustrate the intuition that motivates the theorem. Consider the following one-dimensional model

$$\mathcal{H}_0(k) = 2\cos k \tag{25}$$

placed on a chain of length L. Under the periodic boundary condition, the two Bloch waves e^{ik_0x} and e^{-ik_0x} have the same energy $E(k_0) = 2\cos k_0$. When the system has open boundary condition, the Bloch wave e^{ik_0x} will be reflected to e^{-ik_0x} with a π-phase shift. Their linear superposition $e^{ik_0x} - e^{-ik_0x}$ is an eigenstate with energy $2\cos k_0$ that satisfies the zero boundary condition at $x = 0, L$, thus being an open-boundary eigenstate. When the system is added a momentum-dependent dissipation,

$$\mathcal{H}(k) = 2\cos k + i\sin k, \tag{26}$$

the spectrum $E(k)$ becomes complex and forms an ellipse in the complex plane. In this case, the degeneracy is broken, e.g. $E(k) \neq E(-k)$, which implies the open-boundary eigenstates are no longer the linear superposition of the extended Bloch waves. This implies the emergence of skin effect.

Extend the above arguments to two dimensions, and we can provide a physical explanation for the theorem proved in the Supplementary Note 1.B.

Formally, we consider a single-band model

$$\mathcal{H}(\mathbf{k}) = \mathcal{H}_0(\mathbf{k}) + i\Gamma(\mathbf{k}). \tag{27}$$

When the real and imaginary parts of which are functionally independent, the Hamiltonian will have a non-zero spectral area. For a given eigenvalue E_0 of the Bloch Hamiltonian, by solving $\mathcal{H}_0(\mathbf{k}) = \operatorname{Re} E_0$ and $\Gamma(\mathbf{k}) = \operatorname{Im} E_0$, one can obtain a finite set of preimage of E_0, i.e., $\mathbf{K}(E_0) = \{\mathbf{k}_1, ..., \mathbf{k}_m\}$, which includes all Bloch waves having energy E_0. Now suppose that one of the Bloch waves $\mathbf{k}_i \in \mathbf{K}(E_0)$ is incident on the boundary, depending on the normal direction of the boundary, the corresponding momentum of the reflected wave can be arbitrary. However, the number of elements of $\mathbf{K}(E_0)$ is finite, and as such cannot support so many reflection channels. This failure of reflection mechanism at a generic boundary means the failure in forming an open boundary eigenstate from Bloch waves, which implies the emergence of skin effect under a generic open-boundary geometry. However, the spectrum collapses into an arc (zero spectral area) if the real and imaginary parts of the Hamiltonian are functionally dependent, and the number of the corresponding solutions of $\mathcal{H}(\mathbf{k}) = E_0$ is infinite. It means that there are infinite reflection channels to satisfy the open boundary of any shape, and an open boundary eigenstate can be formed from superimposing all Bloch-wave channels.

Concretely, we choose two examples to demonstrate the above arguments. The first example is

$$\mathcal{H}(\mathbf{k}) = 2\cos k_x + 2i\cos k_y, \tag{28}$$

of which the spectral area is nonzero shown in Fig. 4(a). For a given eigenvalue $E_0 = 1+i$, by solving $2\cos k_x = 1$ and $2\cos k_y = 1$, we can obtain a finite set of pre-images of E_0, that is, $\mathbf{K}(E_0) = \{k_1, k_2, k_3, k_4\}$ [red points in Fig. 4(b)].

The finite solutions of $\mathcal{H}(\mathbf{k}) = E_0$ cannot support so many reflection channels, that is to say, cannot form an open-boundary eigenstate on a generic geometry by superimposing these Bloch waves specified by $k_{i=1,2,3,4}$. Therefore, the Hamiltonian Eq. (28) has skin effect under open-boundary geometry of a generic shape. The second example reads

$$\mathcal{H}(\mathbf{k}) = 2\cos(k_x + k_y) + 2i\cos(k_x + k_y), \tag{29}$$

the periodic-boundary spectrum of which is an arc [the gray line in Fig. 4(c)]. The set of pre-images of $E_0 = 1 + i$ has infinite elements [the red lines in Fig. 4(d)], which means that there are infinite ways of superimposing these Bloch waves to satisfy the open boundary condition of any shape. Therefore, the Hamiltonian Eq. (29) has no skin effect under any open-boundary geometry.

SUPPLEMENTARY NOTE 3: THE CURRENT FUNCTIONAL AND SKIN EFFECT

We divided this section into three parts. First, we give a general mathematical definition of the current functional (including zero and nonzero current functional). Second, we can completely classify the skin effect in two and higher dimensions into two types, that is, non-reciprocal skin effect (NRSE) and generalized reciprocal skin effect (GRSE), according to current functional. Finally, we discuss the restriction of symmetry on the current functional and the compatibility of skin effect with symmetry.

A. The definition of the current functional

In d dimensions, generally, the current functional is defined as

$$J_{\boldsymbol{\alpha}}[n] = \sum_{\mu} \oint_{\mathrm{BZ}} dk^d n(E_\mu, E_\mu^*) \nabla_{\boldsymbol{\alpha}} E_\mu(\mathbf{k}) \tag{30}$$

under the periodic-boundary condition, where μ represents the energy band index and $\nabla_{\boldsymbol{\alpha}}$ is the directional derivative along certain direction $\boldsymbol{\alpha} = \sum_{i=1}^{d} \alpha_i \, \hat{e}_{k_i}$ in d-dimensional momentum space (Here $\hat{e}_{k_i}$ represents the i-th basis in momentum space).

Here, $n(E, E^*)$ represents a distribution function when the system is in a steady state and only depends explicitly on the complex energy of the system state. Therefore, the current functional $J_{\boldsymbol{\alpha}}[n]$ is defined as the function of the distribution function $n(E, E^*)$, and different input $n(E, E^*)$ gives different output $J_{\boldsymbol{\alpha}}[n]$ with fixed $\boldsymbol{\alpha}$. In Hermitian case, the current functional becomes $J_{\boldsymbol{\alpha}}[n] = \sum_\mu \oint_{\mathrm{BZ}} dk^d n(E_\mu) \nabla_{\boldsymbol{\alpha}} E_\mu(\mathbf{k}) = \sum_\mu \oint_{\mathrm{BZ}} dk^d n(E_i) v_{\mu,\boldsymbol{\alpha}}(\mathbf{k}) = 0$, where $v_{\mu,\boldsymbol{\alpha}}(\mathbf{k}) = \sum_{i=1}^{d} \alpha_i \frac{\partial E_\mu(\mathbf{k})}{\partial k_i}$ is the group velocity along direction $\boldsymbol{\alpha}$ in the μ-th energy band. In non-Hermitian systems, the energy E is generally a complex number, therefore, the directional derivative of $E_\mu(\mathbf{k})$ along $\boldsymbol{\alpha}$ in Eq.(30) becomes

$$\nabla_{\boldsymbol{\alpha}} E_\mu(\mathbf{k}) = \sum_{j=1}^{d} \alpha_j \left(\frac{\partial \,\mathrm{Re}\, E_\mu(\mathbf{k})}{\partial k_j} + i \frac{\partial \,\mathrm{Im}\, E_\mu(\mathbf{k})}{\partial k_j} \right), \tag{31}$$

which represents the generalized (complex) velocity in d dimensions.

Specially, in one-dimensional non-Hermitian system, the current functional reduces to

$$J^{\mathrm{1D}}[n] = \sum_\mu J_\mu^{\mathrm{1D}}[n] = \sum_\mu \oint_{\mathrm{BZ}} dk \, n(E_\mu, E_\mu^*) \, \partial_k E_\mu(k), \tag{32}$$

where μ represents the band index. The generalized velocity for μ-th band Eq.(31) becomes

$$\partial_k E_\mu(k) = \partial_k \mathrm{Re}\, E_\mu(k) + i \partial_k \mathrm{Im}\, E_\mu(k), \tag{33}$$

which corresponds to the tangent vector of the μ-th energy band on the complex plane.

Based on the above definition of current functional, we further define the “nonzero current functional” as,

$$\exists \boldsymbol{\alpha}, n; \; J_{\boldsymbol{\alpha}}[n] \neq 0, \tag{34}$$

which is simply labeled as $J \neq 0$. As the complementary set, the "zero current functional" (labeled by $J = 0$) is defined as

$$\forall \boldsymbol{\alpha}, n;\; J_{\boldsymbol{\alpha}}[n] = 0, \tag{35}$$

which means the current functional is zero regardless of the choice of $\boldsymbol{\alpha}$ and $n(E, E^*)$. For example, a Hermitian system always has zero current functional [2].

B. Current functional and the classification of skin effect

The nonzero current functional and zero current functional together constitute a complete set mathematically, and they are mutually exclusive. Based on this, we can completely classify the skin effect according to the zero and nonzero current functional.

1. One-dimensional skin effect

In one dimension, the current functional reduces to Eq.(32). In Ref. [2], the authors claim that for a one-dimensional system without any symmetry, if it has nonzero current functional ($J \neq 0$) in Eq.(34), then the system has skin effect (which is called $\mathbb{Z}$ skin effect), and vice versa. Another type of 1D skin effect — $\mathbb{Z}_2$ skin effect has been reported in Ref. [3]. In this case, the system need to respect the spinful anomalous time-reversal symmetry, namely, $\mathcal{U}_T^\dagger \mathcal{H}^t(k)\mathcal{U}_T = \mathcal{H}(-k)$ with $\mathcal{U}_T\mathcal{U}_T^t = -1$. In fact, we can prove that for the $\mathbb{Z}_2$ skin effect, the system always has zero current functional ($J = 0$) in Eq.(35). A simple proof is present as follows.

This anomalous time-reversal symmetry requires the energy bands always come in pair and satisfy $E_\uparrow(k) = E_\downarrow(-k)$. Correspondingly, for this pair of energy bands, the current functional satisfies $J_\uparrow[n] = -J_\downarrow[n]$. Here $n(E, E^*)$ is invariant under the anomalous time-reversal symmetry, because n only depends on the energy and the complex energy is invariant under this symmetry. Therefore, the current functional for all energy bands Eq.(32) must be sum up to zero, regardless of the distribution function $n(E, E^*)$.

To sum up, the 1D skin effect can be completely classified into $\mathbb{Z}$ skin effect with nonzero current functional ($J \neq 0$) and $\mathbb{Z}_2$ skin effect with zero current functional ($J = 0$). (Note that this classification of skin effect according to the current functional is different from the classification of intrinsic point-gap topology for symmetry class [3].)

2. Two- and higher-dimensional skin effect

The theorem tells us that in two and higher dimensions the system has the universal skin effect, if and only if the spectral area is nonzero. According to the current functional, the universal skin effect can be further classified into non-reciprocal skin effect ($J \neq 0$) and generalized reciprocal skin effect ($J = 0$), as shown in Fig. 3 in the main text. By definition in Eq.(34) and Eq.(35), these two types of skin effect are complete and mutually exclusive.

We call the skin effect with nonzero current functional as non-reciprocal skin effect (NRSE), because this type of skin effect incompatible with the inversion or anomalous time-reversal symmetry [4, 5]. Equivalently, the inversion and anomalous time-reversal symmetry always requires zero current functional (the rigorous proof is provided in the next subsection). Therefore, the NRSE is similar to the $\mathbb{Z}$ skin effect in one dimension, because the $\mathbb{Z}$ skin effect in 1D is forbidden by the inversion or anomalous time-reversal symmetry.

We name the skin effect with zero current functional as generalized reciprocal skin effect (GRSE) based on two reasons. The first reason is that this type of skin effect is compatible with the inversion or anomalous time-reversal symmetry. Note that an analog of GRSE in one dimension is the $\mathbb{Z}_2$ skin effect [3], which can appear when the bulk Hamiltonian respects the spinful anomalous time-reversal symmetry ($\mathcal{T}^2 = -1$). The second reason is stated as follows.

A reciprocal system requires the Hamiltonian to satisfy $\mathcal{H}^t = \mathcal{H}$ ($\mathcal{H}^t(\mathbf{k}) = \mathcal{H}(-\mathbf{k})$ in momentum space) [6], which results in zero current functional. But generally, the zero current functional means more. For example, a Hamiltonian satisfying $\mathcal{H}^t(\mathbf{k}) = \mathcal{H}(\mathbf{k} + \mathbf{k}_\theta)$ ($\mathbf{k}_\theta \neq 0$ and $\mathbf{k}_\theta \neq -2\mathbf{k}$) also gives rise to zero current functional. A simple model Hamiltonian reads $\mathcal{H}(\mathbf{k}) = 2\cos k_x + i \sin k_y$, which satisfies $\mathcal{H}^t(k_x, k_y) = \mathcal{H}(-k_x, \pi - k_y)$ and has zero current functional. It can be seen that the zero current functional includes but is not limited to the reciprocal skin effect [6]. Therefore, we term the type of skin effect with zero current functional as the generalized reciprocal skin effect.

C. Current functional and symmetry

In this subsection, we first investigate the restriction of all point-group symmetries and time-reversal symmetry on the current functional. Meanwhile, we discuss the compatibility of all types of skin effect with these symmetries. Finally, we discuss the relationship between the universal skin effect and symmetry.

1. Current functional under point-group symmetry

In this part, we discuss the restriction of all point groups in three dimensions on the current functional. Here, if a point-group symmetry requires zero current functional, then the non-reciprocal skin effect ($J \neq 0$) can not appear under this symmetry. That is to say, the non-reciprocal skin effect is incompatible with this point-group symmetry. Obviously, the generalized reciprocal skin effect ($J = 0$) is compatible with all point-group symmetries. In what follows, we demonstrate that the non-reciprocal skin effect is only compatible with point groups C_m and $C_{2,3,4,6,2v,3v,4v,6v}$.

As the above definition, the current functional for μ-th band can be expressed as

$$J_{\mu,\boldsymbol{\alpha}}[n] = \oint_{\mathrm{BZ}} dk^d n(E_\mu, E_\mu^*) \nabla_{\boldsymbol{\alpha}} E_\mu(\mathbf{k}) = \sum_{j=1}^{d} \alpha_j \oint_{\mathrm{BZ}} dk^d n(E_\mu, E_\mu^*) \partial_{k_j} E_\mu(\mathbf{k}), \tag{36}$$

where $\nabla_{\boldsymbol{\alpha}} = \sum_{j=1}^{d} \alpha_j \partial_{k_j}$ and d is the dimension of the system. Note that the distribution function $n(E, E^*)$ only depends on the energy of the system state. Therefore, $n(E, E^*)$ is invariant under the point-group operation due to the complex energy being invariant.

Inversion: Consider a system that only has inversion symmetry I, and each band satisfies $E_\mu(\mathbf{k}) = E_\mu(I\,\mathbf{k}) = E_\mu(-\mathbf{k})$. With the inversion symmetry, the current functional for μ-th band becomes

$$\begin{aligned} J_{\mu,\boldsymbol{\alpha}}[n] &= \sum_{j=1}^{d} \alpha_j \oint_{\mathrm{BZ}} dk^d n(E_\mu, E_\mu^*) \partial_{k_j} E_\mu(\mathbf{k}) = \sum_{j=1}^{d} \alpha_j \oint_{\mathrm{BZ}} dk^d n(E_\mu, E_\mu^*) \partial_{-k_j} E_\mu(-\mathbf{k}) \\ &= \sum_{j=1}^{d} \alpha_j \oint_{\mathrm{BZ}} dk^d n(E_\mu, E_\mu^*) \partial_{-k_j} E_\mu(\mathbf{k}) = -\sum_{j=1}^{d} \alpha_j \oint_{\mathrm{BZ}} dk^d n(E_\mu, E_\mu^*) \partial_{k_j} E_\mu(\mathbf{k}) = -J_{\mu,\boldsymbol{\alpha}}[n] = 0. \end{aligned} \tag{37}$$

It means that if the Hamiltonian has only inversion symmetry, the current functional for each band must be zero *regardless of* the choice of $n(E, E^*)$. Equivalently, the non-reciprocal skin effect must vanish in the system with inversion symmetry. Therefore, the non-reciprocal skin effect is *incompatible* with the point groups including inversion symmetry, that is, $C_{i,3i,2h,4h,6h}$, $D_{3d,2h,4h,6h}$, T_h and O_h.

Rotation: Consider a system that is invariant under a point group including rotation operator R, then for m-th energy band $E_\mu(\mathbf{k}) = E_\mu(R\,\mathbf{k})$. Under this rotation symmetry, the current functional becomes (the following derivation temporarily ignores the band index μ for simplicity)

$$\begin{aligned} J_{\boldsymbol{\alpha}}[n] &= \sum_{j=1}^{d} \alpha_j \oint_{\mathrm{BZ}} dk^d n(E, E^*) \partial_{k_j} E(R\mathbf{k}) = \sum_{j=1}^{d} \alpha_j \oint_{\mathrm{BZ}} \det{[J_{\mathbf{k},\mathbf{q}}]} dq^d n(E, E^*) \sum_{i=1}^{d} R_{ij} \partial_{q_i} E(\mathbf{q}) \\ &= \sum_{j=1}^{d} \alpha_j \oint_{\mathrm{BZ}} dq^d n(E, E^*) \sum_{i=1}^{d} R_{ij} \partial_{q_i} E(\mathbf{q}) = \sum_{i=1}^{d} \oint_{\mathrm{BZ}} dq^d n(E, E^*) \sum_{j=1}^{d} \alpha_j R^t_{ji} \partial_{q_i} E(\mathbf{q}) \\ &= \sum_{i=1}^{d} \oint_{\mathrm{BZ}} dk^d n(E, E^*) \sum_{j=1}^{d} \alpha_j R^t_{ji}\, \partial_{k_i} E(\mathbf{k}) = \sum_{i=1}^{d} \oint_{\mathrm{BZ}} dk^d n(E, E^*)\, \alpha_i\, \partial_{k_i} E(\mathbf{k}), \end{aligned} \tag{38}$$

where $\det{[J_{\mathbf{k},\mathbf{q}}]}$ in the first row is the determinant of the Jacobian $J_{\mathbf{k},\mathbf{q}}$ that measures the change of differential volume element under different representations and the sign of $\det{[J_{\mathbf{k},\mathbf{q}}]}$ is positive because the rotational operator preserves orientation. One can always choose an appropriate basis transformation such that $\det{[J_{\mathbf{k},\mathbf{q}}]} = 1$. In addition, since the Brillouin zone has the same symmetry group as the Hamiltonian and rotational operator R does not change the orientation, the integral region BZ is invariant under the point group.

The last equation of Eq. (38) requires $\alpha_i = \sum_{j=1}^{d} \alpha_j R^t_{ji} = \sum_{j=1}^{d} R_{ij}\alpha_j$, which can be represented as

$$R\boldsymbol{\alpha} = \boldsymbol{\alpha}, \tag{39}$$

which means that the direction of $\boldsymbol{\alpha}$ is parallel to the rotational axis of R. Meanwhile, the component of $\boldsymbol{\alpha}$ perpendicular to the rotational axis must be zero. For example, assume that R is a rotation operator that rotates θ along k_z axis ($\theta \neq 0$). The matrix representation of R in momentum space is,

$$R = \begin{pmatrix} \cos\theta & -\sin\theta & 0 \\ \sin\theta & \cos\theta & 0 \\ 0 & 0 & 1 \end{pmatrix}, \tag{40}$$

then Eq. (39) can be represented as the following matrix form,

$$\begin{pmatrix} \cos\theta & -\sin\theta & 0 \\ \sin\theta & \cos\theta & 0 \\ 0 & 0 & 1 \end{pmatrix} \begin{pmatrix} \alpha_x \\ \alpha_y \\ \alpha_z \end{pmatrix} = \begin{pmatrix} \alpha_x \\ \alpha_y \\ \alpha_z \end{pmatrix}, \tag{41}$$

which requires $\alpha_x = \alpha_y = 0$. Therefore, only $J_z[n]$ component is allowed under the rotational symmetry along the z axis.

We conclude that if a point group contains two or more rotations with non-parallel rotational axes, the current functional for each band must be zero. If the point group contains only one rotation, a nonzero current functional along the rotational axis is allowed, thus non-reciprocal skin effect is *compatible* with the point groups including only one rotation, namely $C_{2,3,4,6}$.

Mirror: Similarly, a mirror symmetry requires

$$M\boldsymbol{\alpha} = \boldsymbol{\alpha}, \tag{42}$$

which means the nonzero current functional in the mirror-invariant plane is allowed. Therefore, the non-reciprocal skin effect is compatible with the point groups having only one mirror symmetry, that is, C_m.

Consider the point groups with both of rotation symmetry and mirror symmetry. In this case, the $\boldsymbol{\alpha}$ need to satisfy

$$RM\boldsymbol{\alpha} = \boldsymbol{\alpha}. \tag{43}$$

Therefore, the current functional $J_{\boldsymbol{\alpha}}[n]$ can be nonzero when the rotational axis lies on the mirror plane. The point groups satisfying the above condition include $C_{2v,3v,4v,6v}$. Therefore, the non-reciprocal skin effect is *compatible* with these point groups $C_{2v,3v,4v,6v}$.

So far, we discussed all three-dimensional point groups that allow the current functional to be nonzero. The same procedure can be easily done for the point groups in two dimensions. Equivalently, we can conclude that the non-reciprocal skin effect can appear under (or is compatible with) the following point groups,

$$\{C_m, C_2, C_3, C_4, C_6, C_{2v}, C_{3v}, C_{4v}, C_{6v}\}. \tag{44}$$

2. Current functional with time-reversal symmetry

Before, we examine the restriction of all unitary point groups on the current functional and all types of skin effect. In this part, we simply discuss the current functional and skin effect in the system with time-reversal symmetry.

Here, the time-reversal symmetry refers to the collection of the two different time-reversal symmetries in Ref. [4], namely, the conventional (complex-conjugate type) one $\mathcal{T}$ and the anomalous (transpose type) one $\bar{\mathcal{T}}$. Note that in one dimension, the non-Hermitian skin effect is compatible with $\mathcal{T}$ but incompatible with the spinless anomalous time-reversal symmetry ($\bar{\mathcal{T}}^2 = +1$) [5]. Therefore, only $\bar{\mathcal{T}}$ need to be considered for the higher-dimensional skin effect.

A Hamiltonian respecting the anomalous time-reversal symmetry (aTRS) satisfies

$$\mathcal{T}^{-1}\mathcal{H}(\mathbf{k})\mathcal{T} = \mathcal{U}_T^{\dagger}\mathcal{H}^t(\mathbf{k})\mathcal{U}_T = \mathcal{H}(-\mathbf{k}), \tag{45}$$

where $\mathcal{T}^2 = +1$ refers to the spinless aTRS and $\mathcal{T}^2 = -1$ is the spinful aTRS.

The spinless aTRS requires each energy band to satisfy $E_\mu(\mathbf{k}) = E_\mu(-\mathbf{k})$. As a result, the current functional for each band follows

$$J_{\mu,\boldsymbol{\alpha}}[n] = \oint_{\mathrm{BZ}} dk^d n(E_\mu, E_\mu^*)\nabla_{\boldsymbol{\alpha}} E_\mu(\mathbf{k}) = \oint_{\mathrm{BZ}} dk^d n(E_\mu, E_\mu^*)\nabla_{-\boldsymbol{\alpha}} E_\mu(-\mathbf{k}) = -J_{\mu,\boldsymbol{\alpha}}[n] = 0. \tag{46}$$

However, under the spinful aTRS, the energy bands come in pair and have the restriction, $E_\mu(\mathbf{k}) = E_{\tilde{\mu}}(-\mathbf{k})$, which results in

$$J_{\mu,\boldsymbol{\alpha}}[n] = -J_{\tilde{\mu},\boldsymbol{\alpha}}[n]. \tag{47}$$

In this case, the current functional summing over all bands must be zero. We conclude that the non-reciprocal skin effect is incompatible with the aTRS, while the generalized reciprocal skin effect can appear under this symmetry.

3. The universal skin effect and symmetries

In the previous parts, we analyze the restrictions of all point groups and time-reversal symmetry. If a symmetry requires zero current functional, then the non-reciprocal skin effect is incompatible with this symmetry, which means that the non-reciprocal skin effect must vanish under this symmetry. Instead, as another type of universal skin effect, the generalized reciprocal skin effect can appear under these symmetries. It implies that the universal skin effect can appear under the above discussed symmetries, including all point groups and time-reversal symmetry.

In fact, according to our theorem, the universal skin effect disappears if and only if the spectral area is zero. Therefore, in order to discuss the role of symmetry on the universal skin effect, we need only to consider whether under the symmetry the spectral area is zero or not. However, there are no such internal (or point-group) symmetries to restrict the spectral area to be zero.

Assume that the system has pseudo Hermiticity $\eta\mathcal{H}^\dagger(\mathbf{k})\eta^{-1} = \mathcal{H}(\mathbf{k})$ [4]. The eigenvalues of the Hamiltonian must be real or come in complex-conjugate pairs. It is possible for the system to have entire real Bloch spectrum, under which the spectral area is zero and the universal skin effect disappears. A particular example is $\eta = 1$, which reduces to the Hermitian case. On the other hand, once not all the Bloch spectrum are real but come in complex-conjugate pairs to cover a finite area on the complex plane, the spectral area becomes nonzero, and consequently, the universal skin effect appears.

SUPPLEMENTARY NOTE 4: CORNER-SKIN EFFECT AND GEOMETRY-DEPENDENT-SKIN EFFECT

In this section, we first define the corner-skin effect (CSE) and geometry-dependent-skin effect (GDSE), and discuss the localization of open-boundary eigenstates in CSE and GDSE. In addition, we show some features of the spectrum in GDSE example in the main text, and numerically verify that GDSE obeys the volume law, which differentiates from normal boundary states or higher-order skin modes [7, 8].

A. The corner-skin effect

In this subsection, we define the CSE, and calculate the generalized Brillouin zone of the CSE example (Eq.(2) in the main text) to demonstrate the localization of the open-boundary eigenstates.

1. Definition

We define the CSE as a type of the non-reciprocal skin effect ($J \neq 0$) that exhibits the particular *phenomenon* that almost all eigenstates are localized at corners of the open-boundary geometry.

Therefore, the CSE inherits the features of the non-reciprocal skin effect, including nonzero current functional in Eq.(34) and incompatibility with all point groups except for

$$\{C_m, C_2, C_3, C_4, C_6, C_{2v}, C_{3v}, C_{4v}, C_{6v}\}. \tag{48}$$

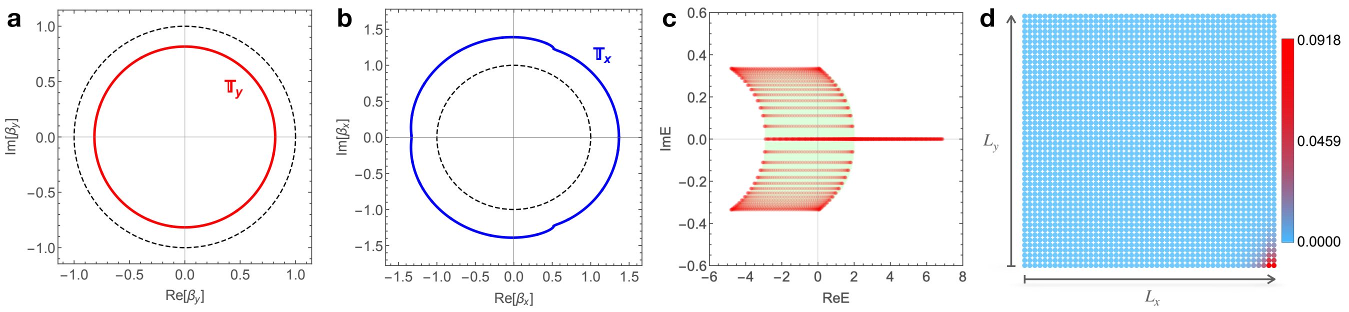

Supplementary Figure 5. The two-dimensional GBZ of Hamiltonian Eq. (50) is the direct product of y-subsystem GBZ [the red circle in (a)] and x-subsystem GBZ [the blue loop in (b)], and for comparison, the Brillouin zone is plotted as the dashed gray unit circle. The 2D GBZ reproduces the open-boundary spectrum under the thermodynamic limit, shown as the light blue region in (c), which is consistent with the numerically calculated eigenvalues (the red points) of the Hamiltonian on a square geometry with system size $L_x = L_y = 50$. In addition, the spatial distribution of eigenstates $W(x)$ is shown in (d).

2. *The localization of eigenstates in CSE*

The precise position where the eigenstates are localized depends on the generalized Brillouin zone (GBZ). As will be explained in the following contents, in the model shown in Fig. 3(c) of the main text, the GBZ can be solved exactly. However, for a generic model with arbitrary open boundary conditions, the calculation of the corresponding GBZ is still an open question.

Now we calculate the GBZ of the model for CSE in Fig. 3(c). We first write down the Hamiltonian of the model

$$\mathcal{H}(\mathbf{k}) = [5(\cos k_x + \cos 2k_x) - i(\sin k_x + 3\sin 2k_x) + 5\cos k_y + i\sin k_y]/2. \tag{49}$$

Notice that under the open boundary of square geometry , the system can be solved by the separation of variables method. By replacing e^{ik_x} and e^{ik_y} with complex variables β_x and β_y, respectively, the Hamiltonian can be rewritten as a polynomial

$$\mathcal{H}(\beta_x, \beta_y) = h_x(\beta_x) + h_y(\beta_y), \tag{50}$$

where

$$h_x(\beta_x) = \frac{\beta_x^2}{2} + \beta_x + \frac{3}{2\beta_x} + \frac{2}{\beta_x^2}; \quad h_y(\beta_y) = \frac{3\beta_y}{2} + \frac{1}{\beta_y}. \tag{51}$$

For the x- and y- directions, the corresponding 1D GBZs are determined by the following characteristic equations

$$h_x(\beta_x) - E_x = 0, \qquad h_y(\beta_y) - E_y = 0, \tag{52}$$

respectively. Using the approach developed in previous Refs. [9–11], the results are shown in Fig. 5 (a) and (b), where $\mathbb{T}_x$ and $\mathbb{T}_y$ denote the GBZs in the x- and y- directions, respectively. Having obtained $\mathbb{T}_x$ and $\mathbb{T}_y$, the corresponding asymptotic energy spectrum of the 2D system in the thermodynamic limit becomes

$$E_{\mathrm{OBC}} = h_x(\beta_x \in \mathbb{T}_x) + h_y(\beta_y \in \mathbb{T}_y), \tag{53}$$

which is shown as the light blue region in Fig. 5 (c).

Now the precise localization position of the eigenstates can be analyzed the corresponding 2D GBZ, which is $\mathbb{T}_x \times \mathbb{T}_y$ in this model. Since the radius of $\mathbb{T}_y$ is less than 1, all eigenstates are localized in the negative y-direction. In addition, the radius of $\mathbb{T}_x$ greater than 1 indicates that all eigenstates concentrate in the positive x-direction. Therefore, the open-boundary eigenstates are localized in the lower right corner of the square geometry, as shown in Fig. 5(d).

3. *Volume Law in CSE*

As shown in Fig. 6(a), we choose the block enclosed by the dashed blue line as the corner region, denoted as $\mathcal{R}_C$. In this two-dimensional square lattice, $\mathcal{R}_C$ is specified as the $9 * 9$ lattice sites at the right lower corner. Meanwhile,

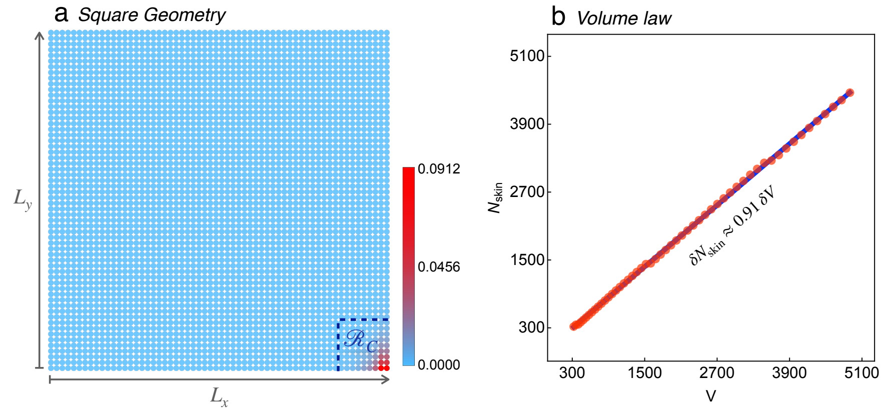

Supplementary Figure 6. The spatial distribution of $W(\mathbf{x})$ in the CSE model Hamiltonian [Eq.(2) in the main text] is plotted in (a) with the colorbar, where the system size of the square lattice is $L_x = L_y = 60$ and the corner region $\mathcal{R}_C$ refers to the $9*9$ sites at the right lower corner. (b) The red dots are the raw data and the blue line is the fitting curve with $\delta N_{\text{skin}} \approx 0.91\delta V$, which shows the volume law in CSE.

we define the quantity

$$W(\mathbf{x}) = \frac{1}{N}\sum_n |\psi_n(\mathbf{x})|^2, \tag{54}$$

where $\psi_n(\mathbf{x})$ is the n-th normalized right eigenstate and N is the number of these eigenstates [$N = 60*60 = 3600$ in Fig. 6(a)]. We immediately know that the sum of $W(\mathbf{x})$ over the entire lattice sites must be 1, or symbolically,

$$\sum\nolimits_{\mathbf{x}\in\text{Lat}} W(\mathbf{x}) = 1, \tag{55}$$

where "Lat" symbolically refers to the set of all lattice sites.

For each given Hamiltonian, we can plot the spatial distribution of $W(\mathbf{x})$ and mark the value of $W(\mathbf{x})$ in different colors. This map is reflected in the colorbar, as shown in Fig. 6(a). If the system has no skin effect, the value of $W(\mathbf{x})$ at each site is basically uniform and approximately equal to $1/N$. However, in the system with CSE [Eq.(2) in the main text], $W(\mathbf{x})$ is inhomogeneous in the square lattice, and, as shown in the colorbar of Fig. 6(a), the maximum of $W(\mathbf{x})$ is about 0.0912. Here, we sum $W(\mathbf{x})$ over the right lower corner region $\mathcal{R}_C$ and obtain

$$\sum\nolimits_{\mathbf{x}\in\mathcal{R}_C} W(\mathbf{x}) = 0.9551 \gg \sum\nolimits_{\mathbf{x}\in\mathcal{R}_C} \frac{1}{N} = 0.0225. \tag{56}$$

Note that the sum of $W(\mathbf{x})$ over the right lower corner region is close to 1, which reveals that almost all eigenstates in CSE concentrate in the corner region $\mathcal{R}_C$.

In addition, we take a scaling analysis for the CSE model. First, we judge an eigenstate $\psi_i(\mathbf{x})$ as a corner mode when it satisfies the following condition,

$$\sum_{x\in\mathcal{R}_C} |\psi_i(\mathbf{x})|^2 \geq 90\%, \tag{57}$$

which means that we can find this mode with 90% probability in the corner region $\mathcal{R}_C$. Here, the first point is that $\mathcal{R}_C$ always refers to the $9*9$ sites at the right lower corner of the square lattice regardless of the lattice size. The second point is that 90% here is not a rigid criterion for judging the corner skin mode, while what truly matters is the volume law.

Second, we count the number of corner skin modes as increasing the lattice size from $L = 18$ to $L = 70$ ($V = L^2$). The data has been plotted in Fig. 6(b), where the red dots are the raw data and the blue line is the fitting curve with

$$\delta N_{\text{skin}} \approx 0.91\delta V, \tag{58}$$

which tells us the corner skin modes follow the volume law.

B. The geometry-dependent-skin effect

In this subsection, we define the GDSE and give a criterion to determine where the open-boundary eigenstates are localized in the system with GDSE. Then we show some features in the spectra of the GDSE example (Eq.(3) in the main text). Finally, we take a numerical verification of the volume law.

1. Definition

Similar to the definition of CSE, the GDSE is one type of generalized reciprocal skin effect ($J = 0$) showing the unique *phenomenon* that there is at least one fully open boundary geometry under which the skin effect does not appear.

Equivalently, the system exhibits GDSE when the system satisfies the following two points at the same time, (i) the spectral area is nonzero; (ii) there is at least one fully open boundary geometry under which the skin effect does not appear.

Therefore, GDSE inherits the features of the generalized reciprocal skin effect, including the zero current functional in Eq.(35) and compatibility with all point-group symmetries.

According to the definition of GDSE, the skin effect will disappear in certain open-boundary geometries. In what follows, we take an example with mirror symmetry to introduce an efficient method to find the geometry not showing the skin effect.

If the bulk Hamiltonian $\mathcal{H}(\mathbf{k})$ has one mirror symmetry, e.g., the mirror-x symmetry $\mathcal{M}_x\mathcal{H}(k_x,k_y)\mathcal{M}_x^{-1} = \mathcal{H}(-k_x,k_y)$, the (vertical) boundaries parallel to the mirror line does not exhibit the skin effect due to the spectral winding number being zero,

$$\begin{aligned} w_{E_b}(k_y) &= \frac{1}{2\pi i}\int_{-\pi}^{\pi} dk_x\partial_{k_x}\log\det[\mathcal{H}(k_x,k_y)-E_b] = \frac{1}{2\pi i}\int_{-\pi}^{\pi} dk_x\partial_{-k_x}\log\det[\mathcal{H}(-k_x,k_y)-E_b] \\ &= \frac{1}{2\pi i}\int_{-\pi}^{\pi} dk_x\partial_{-k_x}\log\det[\mathcal{M}_x\mathcal{H}(k_x,k_y)\mathcal{M}_x^{-1}-E_b] = -w_{E_b}(k_y) = 0, \end{aligned} \tag{59}$$

regardless of the reference energy E_b. If the bulk Hamiltonian has another mirror symmetry, for example, the mirror-y symmetry $\mathcal{M}_y\mathcal{H}(k_x,k_y)\mathcal{M}_y^{-1} = \mathcal{H}(k_x,-k_y)$, the (horizontal) boundaries parallel to the mirror-y line does not show the skin effect for the same reason. Therefore, we conclude that if the bulk Hamiltonian has two or more mirror symmetries, the skin effect does not appear on the open-boundary geometry with each boundary parallel to one of these mirror lines (one example is Fig. 3(g) in the main text).

2. The localization of eigenstates in GDSE

In this part, we provide a method to determine which edge (or surface) the eigenstates are localized on for a given open-boundary geometry.

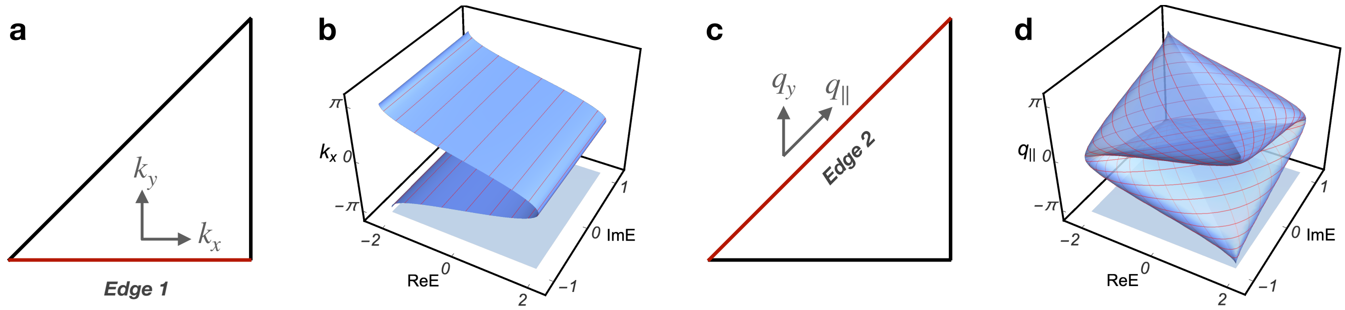

Supplementary Figure 7. To probe the skin effect on edge 1 and edge 2 of the triangle geometry, one needs to reselect the corresponding momentum basis as shown in (a) and (c), respectively. The spectra of the Hamiltonian Eq.(63) in E-k_x and E-$q_\parallel$ space are plotted in (b) and (d), respectively, where the blue region represents the spectral projection onto the complex energy plane.

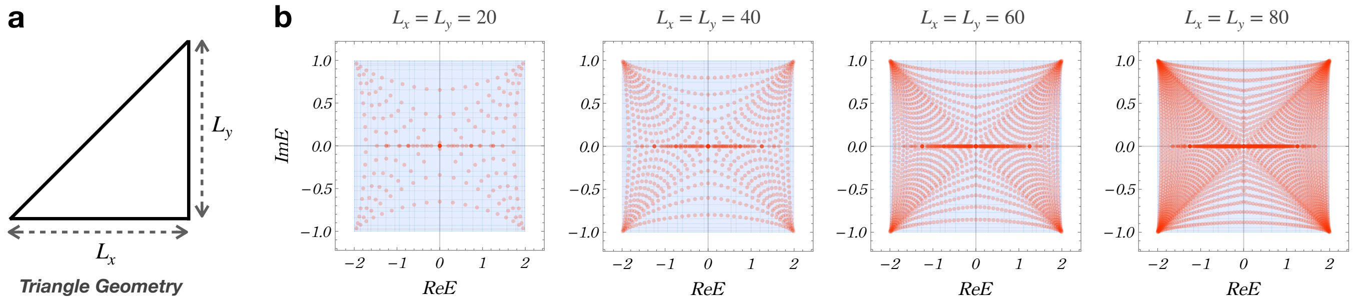

Supplementary Figure 8. (a) The triangle geometry. (b) The red points represent the eigenenergies of the Hamiltonian $\mathcal{H}$ (Eq.(3) in the main text) under triangle geometry with different system size; the light blue area is the periodic-boundary spectrum $\mathcal{H}(\mathrm{BZ})$.

Now we define the winding number. In two dimensions, for each edge of the open-boundary geometry, we first transform the momentum basis from $\mathbf{k} = (k_x, k_y)^T$ to $\mathbf{q} = (q_\parallel, q_y)^T$ by

$$\mathbf{q} = S\mathbf{k}; \quad \det S = 1. \tag{60}$$

Here, $q_\parallel$ is chosen as the momentum parallel to the edge, and S is a $2 * 2$ matrix with unitary determinant, which is the element of group $\mathrm{SL}_2(\mathbb{Z})$. Note that the choice of q_y is not unique, it only needs to satisfy Eq.(60). Accordingly, the Hamiltonian transforms from $\mathcal{H}(k_x, k_y)$ to $\tilde{\mathcal{H}}(q_\parallel, q_y)$. Based on the above definition, we state that if the following condition is satisfied,

$$\forall q_\parallel \in [-\pi, \pi], \quad \forall E_b \in \mathbb{C}, \qquad w_{E_b}(q_\parallel) = \frac{1}{2\pi i}\int_{-\pi}^{\pi} dq_y \partial_{q_y} \ln\det[\tilde{\mathcal{H}}(q_\parallel, q_y) - E_b] = 0, \tag{61}$$

then, no eigenstates are localized at this edge parallel to $q_\parallel$. On the contrary, if the above condition is destroyed, some eigenstates must be localized at the corresponding edge. Note that this statement is strictly true when the other directions are periodic, and it is our conjecture in Fig. 2(a) when other directions are open.

Now we take the single-band example of GDSE (the example Eq.(3) in the main text) to explain it in more detail.

The bulk Hamiltonian reads

$$\mathcal{H}(\mathbf{k}) = 2\cos k_x + i\cos k_y. \tag{62}$$

For the "edge 1" and "edge 2" of the triangle geometry in Fig. 7(a)(c), the transformation matrix S equal to $\{\{1,0\},\{0,1\}\}$ and $\{\{1,1\},\{0,1\}\}$, respectively. Obviously, for any fixed k_x, the spectral winding number $w_{E_b}(k_x) = 0$, as shown in Fig. 7(b). The zero spectral winding number is guaranteed by the mirror symmetry in y direction of the bulk Hamiltonian, $\mathcal{H}(k_x, k_y) = \mathcal{H}(k_x, -k_y)$. For "edge 2" of the geometry, the Hamiltonian can be transformed into

$$\tilde{\mathcal{H}}(\mathbf{q}) = 2\cos(q_\parallel - q_y) + i\cos q_y. \tag{63}$$

For the fixed $q_\parallel$, the spectrum of $\tilde{\mathcal{H}}(\mathbf{q})$ forms an closed loop on the complex plane, as shown in Fig. 7(d). The above process can be taken for every edge of the open-boundary geometry. Therefore, the eigenstates are localized on the "edge 2" instead of "edge 1" of the triangle geometry, as shown in Fig. 3(h) of the main text.

3. The feature of spectrum in the GDSE example

We calculate the eigenstates and spectra of Hamiltonian Eq.(62) on the triangle geometry with different system size. From Fig. 8 we can observe that as the system size increases, the area covered by the open-boundary spectrum on the complex plane becomes gradually larger. It can be excepted that the area of the open-boundary spectrum under the thermodynamic limit tend to be the same as the spectral area (the light blue region in Fig. 8).

Note that even if the area of the energy spectrum under some open-boundary geometry seems to be the same as the spectral area, the system still has the skin effect due to the different density of states on the complex-energy plane, which is a unique feature in two- and higher-dimensional skin effect distinct from the one-dimensional case.

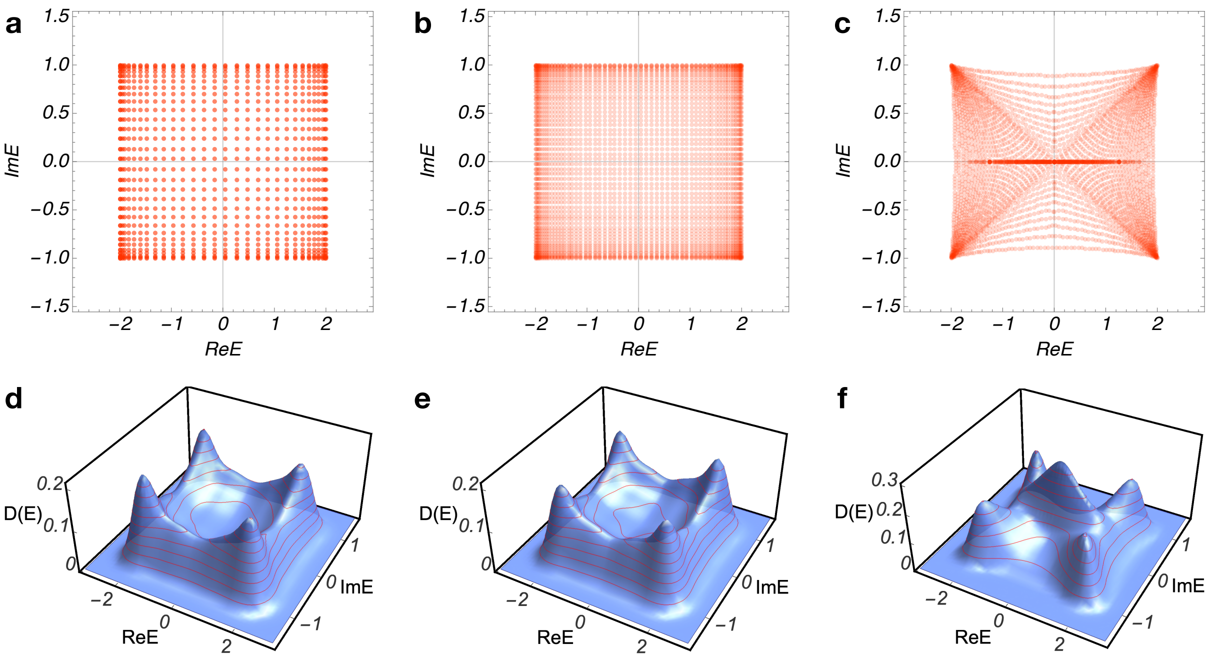

Supplementary Figure 9. The energy spectra of the Hamiltonian (Eq.(3) in the main text) under periodic boundary condition, square and triangle geometry are shown in (a-c), respectively, and the corresponding density of states on the complex plane is shown in (d-f). In (a)(d), the 60*60 **k**-grid is used. In (b)(e) and (c)(f), the system size under square geometry (geometry 1 in Fig. 3 of the main text) and triangle geometry (geometry 2 in Fig. 3 of the main text) is taken as $L_x = L_y = 60$ and $L_x = L_y = 85$, respectively.

We show the density of states plot to support the statement about GDSE in the main text. In Fig. 9, the open-boundary spectra under periodic boundary, square open-boundary geometry and triangle open-boundary geometry are shown in Fig. 9(a)(b)(c), respectively. The corresponding density of states on the complex energy plane are plotted in Fig. 9(d-f), where the z-axis $D(E)$ indicates the probability density of eigenvalues lying in the unit energy interval at E on the complex plane. The spectra under periodic boundary condition and square geometry have the same density of states on the complex energy plane as shown in Fig. 9(d)(e), where the spectral distribution at the corner is denser than the center. It is not the case on the triangle geometry. Even though the same region is covered by their spectra, the eigenvalues under the triangle geometry are more densely distributed at the center of the spectra as shown in Fig. 9(f).

4. Volume law in GDSE

We numerically verify that the geometry-dependent-skin effect of the model in the main text follows the volume law, that is, the increase in the number of skin modes is proportional to the increase in volume of the system,

$$\delta N_{skin} \propto \delta V. \tag{64}$$

Here, we judge an eigenstate $\psi(\mathbf{x})$ as a skin mode when it satisfies

$$\sum_{\mathbf{x}\in\mathcal{B}} |\psi(\mathbf{x})|^2 \geq 90\%, \tag{65}$$

where $\mathcal{B}$ represents the boundary region that we specify.

The Hamiltonian of the tight-binding model for GDSE is $\mathcal{H}(k_x, k_y) = 2\cos k_x + i\cos k_y$. The spatial distribution $W(\mathbf{x})$ of the eigenstates of this Hamiltonian under different open boundaries are plotted in Fig. 10(a)(b). One can observe that the skin modes disappear under the square geometry, but reappear under the parallelogram geometry, which is the feature of GDSE. For the parallelogram geometry, we specify the thickness of the boundary to be the width of three unit cells, and use the blue dashed lines to distinguish the boundary from the bulk in Fig. 10(b). If

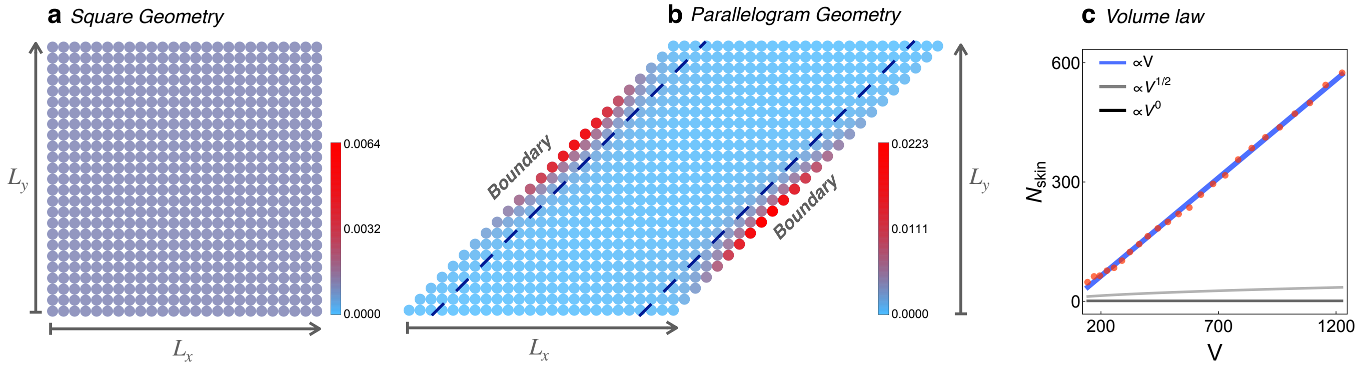

Supplementary Figure 10. The spatial distribution $W(\mathbf{x})$ of the eigenstates of the Hamiltonian (Eq.(3) in the main text) under the square geometry (a) and the parallelogram geometry (b) are plotted. The system size is taken as $L_x = L_y = 25$. The data of the number of skin modes N_{skin} under different system size $V = L_x \times L_y$ is marked by red dots in (c). The volume law is verified by fitting these data into the blue line where $\delta N_{\text{skin}} \approx 0.494\,\delta V$.

an eigenstate of the Hamiltonian under the parallelogram geometry satisfies Eq.(65), we count it as a skin mode. We count the number of skin modes N_{skin} for different system size ($V = L_x \times L_y$), and fit the data (the red dots) into the blue line in Fig. 10(c). The results show that the GDSE satisfies the volume law, specifically, $\delta N_{\text{skin}} \approx 0.494\,\delta V$ in this model.

SUPPLEMENTARY NOTE 5: THE COROLLARY OF THE THEOREM

In this section, we will prove the corollary of our theorem, that is, all stable exceptional semimetals imply the universal skin effect. We review the topological charge of non-Hermitian band degeneracy in the first two subsection. Finally, we obtain the corollary of the theorem and prove the statement in the main text that the stable exceptional points for two-band model indicate nonzero spectral area $A_{\pm}$.

A. Non-Hermitian band degeneracy

Consider a general m-band non-Hermitian Bloch Hamiltonian (with periodic boundary condition),

$$\mathcal{H}(\mathbf{k}) = \sum_{s=1}^{m^2-1} \left[h_s^r(\mathbf{k}) + i h_s^i(\mathbf{k})\right] \Gamma_s, \tag{66}$$

where Γ_s are the generators of Lei algebra $\mathfrak{su}(m)$ and $h_s^r(\mathbf{k})$ and $h_s^i(\mathbf{k})$ are real functions of $\mathbf{k}$. When $m = 2, 3, 4$, Γ_s refer to the Pauli, Gell-Mann, and Dirac matrices, respectively. The eigenvalues of $\mathcal{H}(\mathbf{k})$ can be obtained by solving the following characteristic polynomial

$$f_E(\mathbf{k}) = \det[E - \mathcal{H}(\mathbf{k})] = \prod_{i=1}^{m} [E - E_i(\mathbf{k})], \tag{67}$$

where $E_i(\mathbf{k})$ is the ith eigenvalue of the non-Hermitian Hamiltonian $\mathcal{H}(\mathbf{k})$. At the degeneracy point $\mathbf{k}_D$, two bands must have the same energy, i.e.

$$E_i(\mathbf{k}_D) = E_j(\mathbf{k}_D) \tag{68}$$

for some $i \neq j$. In Ref. [12, 13], the authors have shown that the above condition is equivalent to the vanishing of the discriminant of $f_E(\mathbf{k})$, i.e.

$$\text{Disc}_E[\mathcal{H}](\mathbf{k}_D) = 0, \tag{69}$$

where

$$\mathrm{Disc}_E[\mathcal{H}](\boldsymbol{k}) = \prod_{i<j} [E_i(\boldsymbol{k}) - E_j(\boldsymbol{k})]^2 \tag{70}$$

is the discriminant of $f_E(\mathbf{k})$. Although the discriminant is defined by the roots of $f_E(\mathbf{k}) = 0$, it can be computed directly from the determinant of the Sylvester matrix of $f_E(\mathbf{k})$ and $\partial_E f_E(\mathbf{k})$, which can be expressed by the coefficients of $f_E(\mathbf{k})$. Now we take a generic two-band model as an example to demonstrate this.

Example: Consider a generic two-band model

$$\mathcal{H}(\mathbf{k}) = h_0(\mathbf{k})\sigma_0 + h_x(\mathbf{k})\sigma_x + h_y(\mathbf{k})\sigma_y + h_z(\mathbf{k})\sigma_z, \tag{71}$$

where $h_\mu(\mathbf{k}) = h_\mu^r(\mathbf{k}) + i h_\mu^i(\mathbf{k})$ are complex functions of $\mathbf{k}$. The characteristic polynomial of the two-band model can be written as

$$f_E(\mathbf{k}) = E^2 + b(\mathbf{k})E + c(\mathbf{k}), \tag{72}$$

with $b(\mathbf{k}) = -2h_0(\mathbf{k})$ and $c(\mathbf{k}) = h_0^2(\mathbf{k}) - h_x^2(\mathbf{k}) - h_y^2(\mathbf{k}) - h_z^2(\mathbf{k})$. Computing the discriminant of polynomial (72) with respect to the energy E, we obtain the following condition for the existence of degeneracy points

$$\mathrm{Disc}_E[\mathcal{H}](\mathbf{k}) = b^2(\mathbf{k}) - 4c(\mathbf{k}) = 4[h_x^2(\mathbf{k}) + h_y^2(\mathbf{k}) + h_z^2(\mathbf{k})] = 0. \tag{73}$$

This condition can also be obtained from the energy spectrum, that is, the two bands $E_\pm = h_0(\mathbf{k}) \pm ([h_x^2(\mathbf{k}) + h_y^2(\mathbf{k}) + h_z^2(\mathbf{k}))^{1/2}$ have crossings, where the square root vanishes.

From the above example, one can notice that the discriminant $\mathrm{Disc}_E[\mathcal{H}](\mathbf{k})$ is a complex periodic function of $\mathbf{k}$. Its vanishing requires the real and imaginary parts to be zero at the same time, i.e.

$$\mathrm{Re}\,\mathrm{Disc}_E[\mathcal{H}](\mathbf{k}) = \mathrm{Im}\,\mathrm{Disc}_E[\mathcal{H}](\mathbf{k}) = 0. \tag{74}$$

The solutions of the above equation correspond to the non-Hermitian degeneracy points in 2D and lines in 3D.

B. Topological charge of non-Hermitian band degeneracy

In this subsection, we will review the topological charge of the non-Hermitian band degeneracies. Based on the discriminant of the characteristic polynomial, one can define the topological charge of the degeneracy point $\mathbf{k}_D$, i.e.

$$\nu\left(\mathbf{k}_D\right) = \frac{1}{2\pi i} \oint_{\Gamma(\mathbf{k}_D)} d\mathbf{k} \cdot \nabla_{\mathbf{k}} \ln \mathrm{Disc}_E[\mathcal{H}](\mathbf{k}), \tag{75}$$

where $\Gamma\left(\mathbf{k}_D\right)$ is a loop encircling the degeneracy point $\mathbf{k}_D$. Since $\mathrm{Disc}_E[\mathcal{H}](\mathbf{k})$ is single valued, this invariant is quantized, which is called the discriminant number in Ref. [13]. Putting

$$\mathrm{Disc}_E[\mathcal{H}](\mathbf{k}) = \prod_{i<j} [E_i(\mathbf{k}) - E_j(\mathbf{k})]^2 \tag{76}$$

into $\nu(\mathbf{k}_D)$, one can obtain

$$\begin{aligned}
\nu(\mathbf{k}_D) &= \frac{1}{2\pi i} \oint_{\Gamma(\mathbf{k}_D)} d\mathbf{k} \cdot \nabla_{\mathbf{k}} \ln \prod_{1\le i<j\le n} [E_i(\boldsymbol{k}) - E_j(\boldsymbol{k})]^2 \\
&= \frac{1}{2\pi i} \sum_{i\neq j} \oint_{\Gamma(\mathbf{k}_D)} d\mathbf{k} \cdot \nabla_{\mathbf{k}} \ln [E_i(\boldsymbol{k}) - E_j(\boldsymbol{k})] \\
&= \frac{1}{2\pi} \sum_{i\neq j} \oint_{\Gamma(\mathbf{k}_D)} d\mathbf{k} \cdot \nabla_{\mathbf{k}} \arg [E_i(\boldsymbol{k}) - E_j(\boldsymbol{k})] \,.
\end{aligned} \tag{77}$$

Therefore, for a two-band system,

$$\nu(\mathbf{k}_D) = \frac{1}{\pi} \oint_{\Gamma(\mathbf{k}_D)} d\mathbf{k} \cdot \nabla_{\mathbf{k}} \arg [E_+(\boldsymbol{k}) - E_-(\boldsymbol{k})] \tag{78}$$

which describes the winding of the complex energy between two bands. Now we show a concrete example of the winding number.

Example: Consider the following low energy Hamiltonian around $\mathbf{k}_D$,

$$\mathcal{H}_1(\delta\mathbf{k}) = \sigma_+ + (\delta k_x + i\delta k_y)\sigma_-, \tag{79}$$

where $\delta\mathbf{k} = \mathbf{k} - \mathbf{k}_D$ and $\sigma_\pm = (\sigma_x \pm i\sigma_y)/2$. The eigenvalues of $\mathcal{H}_1(\delta\mathbf{k})$ are

$$E_\pm(\delta\mathbf{k}) = \pm\sqrt{\delta k_x + i\delta k_y}. \tag{80}$$

When $\mathbf{k} = \mathbf{k}_D$, correspondingly $\delta k_x = \delta k_y = 0$, it can be found that $E_+(\delta\mathbf{k} = 0) = E_-(\delta\mathbf{k} = 0) = 0$. This means $\mathbf{k}_D$ is a non-Hermitian degeneracy point. We rewrite Eq. (80) in polar coordinates as

$$E_\pm(\delta\mathbf{k}) = \pm\delta k_r^{1/2} e^{i\theta/2}, \qquad \theta \in (-\pi, \pi], \tag{81}$$

with $(\delta k_x, \delta k_y) = \delta k_r(\cos\theta, \sin\theta)$. It can be found that $E_+(\delta\mathbf{k})$ and $E_-(\delta\mathbf{k})$ jointly form a spectral loop enclosing the degenerate energy $E_+(\mathbf{k}_D) = E_-(\mathbf{k}_D) = 0$ in the complex plane. The winding number $\nu(\mathbf{k}_D) = 1$ calculated from Eq. (78) captures the topological property of degeneracy points.

The topological charge $\nu(\mathbf{k}_D)$ can be used to classify the non-Hermitian degeneracy points. However, the classification is not complete. As a comparison with $\mathcal{H}_1(\delta\mathbf{k})$, we consider the following low energy Hamiltonian,

$$\mathcal{H}_2(\delta\mathbf{k}) = (\delta k_x + i\delta k_y)^2\sigma_+ + (\delta k_x - i\delta k_y)\sigma_-. \tag{82}$$

Obviously, $\delta\mathbf{k} = 0$ is a degeneracy point. One can further prove that its topological charge is $+1$, which is equal to the charge of $\delta\mathbf{k} = 0$ in $\mathcal{H}_1(\delta\mathbf{k})$. However, these two degeneracy points have different properties, for example,

$$\mathcal{H}_1(\delta\mathbf{k} = 0) = \sigma_+, \qquad \mathcal{H}_2(\delta\mathbf{k} = 0) = 0. \tag{83}$$

One can notice that $\mathcal{H}_1(\delta\mathbf{k} = 0)$ is non-diagonal. This type of non-Hermitian degeneracy points are called exceptional points (EPs). In Ref. [13], the authors have shown that only the exceptional points with $\nu(\mathbf{k}_D) = \pm 1$ are robust in 2D, while any other non-Hermitian band degeneracy points are unstable against non-Hermitian perturbations.

C. Stable exceptional points necessitate nonzero spectral area

Our corollary of the theorem is that all lattice Hamiltonians having stable exceptional points have universal skin effect. Here the stable exceptional point refers to the exceptional point of which the topological charge (discriminant number in Eq.(75)) is ± 1. For the stable exceptional point, the spectral area A_i must be nonzero. This fact is guaranteed by their topological properties. Therefore, the corollary can be obtained according to our theorem.

For simplicity, we here only consider a two-band model, and the Bloch Hamiltonian can be written as

$$\mathcal{H}(\mathbf{k}) = \sum_{i=x,y,z} h_i(\mathbf{k})\sigma_i, \tag{84}$$

where $h_i(\mathbf{k}) = h_i^r(\mathbf{k}) + ih_i^i(\mathbf{k})$. Here we have omitted the $h_0(\mathbf{k})\sigma_0$ term, which is irrelevant to the discussion of exceptional points. The eigenvalues of the Hamiltonian is

$$E_\pm(\mathbf{k}) = \pm\sqrt{\Delta(\mathbf{k})} = \pm\sqrt{h_x^2(\mathbf{k}) + h_y^2(\mathbf{k}) + h_z^2(\mathbf{k})}. \tag{85}$$

The emergence of the exceptional point $\mathbf{k}_{\mathrm{EP}}$ requires that

$$\Delta(\mathbf{k}_{\mathrm{EP}}) = h_x^2(\mathbf{k}_{\mathrm{EP}}) + h_y^2(\mathbf{k}_{\mathrm{EP}}) + h_z^2(\mathbf{k}_{\mathrm{EP}}) = 0 \tag{86}$$

and there exists an invertible matrix P such that

$$P^{-1}\mathcal{H}(\mathbf{k}_{\mathrm{EP}})\,P = \begin{pmatrix} 0 & a \\ 0 & 0 \end{pmatrix} \tag{87}$$

with $a \neq 0$ a general complex number. The topological charge of the exceptional point is

$$\nu(\mathbf{k}_{\mathrm{EP}}) = \frac{1}{2\pi i}\oint_{\Gamma(\mathbf{k}_{\mathrm{EP}})} d\mathbf{k}\cdot\nabla_{\mathbf{k}}\ln\Delta(\mathbf{k}). \tag{88}$$

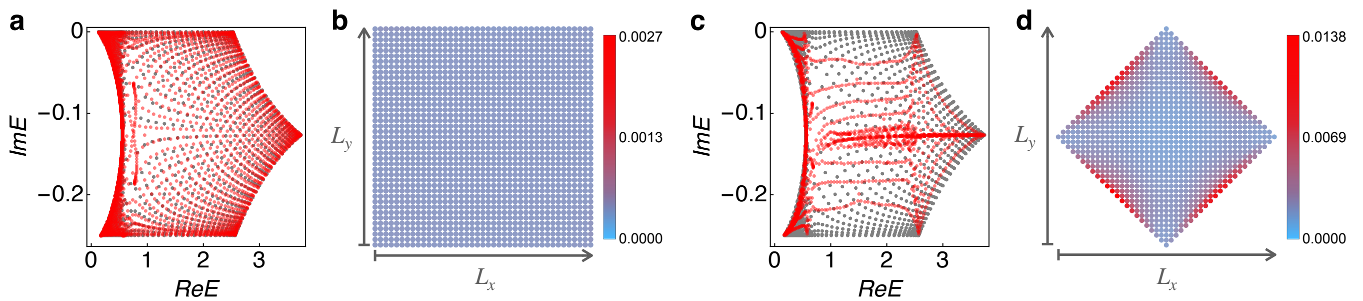

Supplementary Figure 11. The periodic-boundary spectrum of the Hamiltonian Eq.(92) is shown in (a)(c) in gray dots, where $\mathbf{k}$-grid 58×58 is used. Under the square geometry with the systems size $L_x = L_y = 31$, the eigenvalues (the red dots) and the spatial distribution $W(\mathbf{x})$ in Eq.(2) are plotted in (a) and (b), respectively. Under the diamond geometry with the systems size $L_x = L_y = 45$, the eigenvalues (the red dots) and corresponding $W(\mathbf{x})$ are plotted in (c) and (d), respectively.

which describes the winding number of the Bloch spectrum around $E_0 = E_\pm(\mathbf{k}_{\rm EP}) = 0$ and $\Gamma(\mathbf{k}_{\rm EP})$ represents a counterclockwise path enclosing $\mathbf{k}_{\rm EP}$. Thus one can imagine that when $\nu(\mathbf{k}_{\rm EP}) \neq 0$, the corresponding spectral area must be nonzero. Here we note that the above derivation does not require that the degeneracy point is an exceptional point. Actually, any degeneracy point with nonzero topological charge means the nonzero spectral area.

Now we consider the two-dimensional system with stable exceptional points (that is $\nu(\mathbf{k}_{\rm EP}) = \pm 1$). The dispersion around the exceptional point $\mathbf{k}_{\rm EP}$ can be expanded [14] as

$$E_\pm(\mathbf{q}) = \pm\sqrt{c_x\, q_x + c_y\, q_y} + O(|\mathbf{q}|), \tag{89}$$

where c_x, c_y are nonzero complex numbers and $c_{x/y} = c^r_{x/y} + i c^i_{x/y}$ with the superscript r/i indicates the real/imaginary part. The above equation implies that

$$\Delta(\mathbf{q}) \simeq c_x\, q_x + c_y\, q_y = c_x\, \tilde{\Delta}(\mathbf{q}) = c_x(q_x + c_1 q_y) \tag{90}$$

with $c_1 = c_y/c_x = c^r_1 + i\, c^i_1$, where $\tilde{\Delta}(\mathbf{q}) = \mathrm{Re}\,\tilde{\Delta}(\mathbf{q}) + i\,\mathrm{Im}\,\tilde{\Delta}(\mathbf{q}) = (q_x + c^r_1\, q_y) + i\, c^i_1\, q_y$. Putting this equation into the topological charge formula Eq.(88), equivalently, one can obtain that

$$\nu\,(\mathbf{k}_{\rm EP}) = \mathrm{sgn}(\det\begin{bmatrix} \partial_{q_x}\mathrm{Re}\,\tilde{\Delta}(\mathbf{q}) & \partial_{q_y}\mathrm{Re}\,\tilde{\Delta}(\mathbf{q}) \\ \partial_{q_x}\mathrm{Im}\,\tilde{\Delta}(\mathbf{q}) & \partial_{q_y}\mathrm{Im}\,\tilde{\Delta}(\mathbf{q}) \end{bmatrix}) = \mathrm{sgn}(\det\begin{bmatrix} 1 & c^r_1 \\ 0 & c^i_1 \end{bmatrix}) = \mathrm{sgn}(c^i_1), \tag{91}$$

where $\mathrm{sgn}(c^i_1)$ represents the sign of the c^i_1. Note that in our statement in the "Corollary" section of the main text, $c_0 = \sqrt{c_x}$ and $c_1 = c_y/c_x$. Therefore, a stable EP ($\nu(\mathbf{k}_{\rm EP}) = \pm 1$) requires a nonzero imaginary part of c_1, which further ensures a nonzero spectral area $A_\pm$.

SUPPLEMENTARY NOTE 6: THE PHOTONIC CRYSTAL MODEL

In this section, we supplement the spectra and eigenstates of the photonic crystal model [Eq.(4) in the main text]. Then we stack the 2D photonic crystal model along z axis and obtain a three-dimensional example that shows the GDSE. Finally, we explain the anomalous wave-packet dynamics shown in Fig 4(d) of the main text, and comment on the time evolution under the non-Hermitian setting.

A. The spectra and eigenstates of the photonic crystal model

The Hamiltonian of the photonic crystal model reads

$$\mathcal{H}(\mathbf{k}) = \mathbf{d}(\mathbf{k})\cdot\boldsymbol{\sigma} - i\gamma/2(\sigma_0 - \sigma_z), \tag{92}$$

where $\boldsymbol{\sigma} = (\sigma_0, \sigma_x, \sigma_y, \sigma_z)$ is a vector of the Pauli matrices and $\mathbf{d}(\mathbf{k})$ is a vector with four components, that is, $\mathbf{d}(\mathbf{k}) = \{\mu_0 - (t_2 + t_3)(\cos k_x + \cos k_y), t_1[1 - \cos k_x - \cos k_y + \cos(k_x - k_y)], t_1[\sin k_x - \sin k_y - \sin(k_x - k_y)], \mu_z + (t_2 - t_3)(\cos k_x - \cos k_y)\}$. The parameters are chosen as follows, $(t_1, t_2, t_3, \mu_0, \mu_z) = (0.4, -0.1, 0.5, 1.35, -0.02)$.

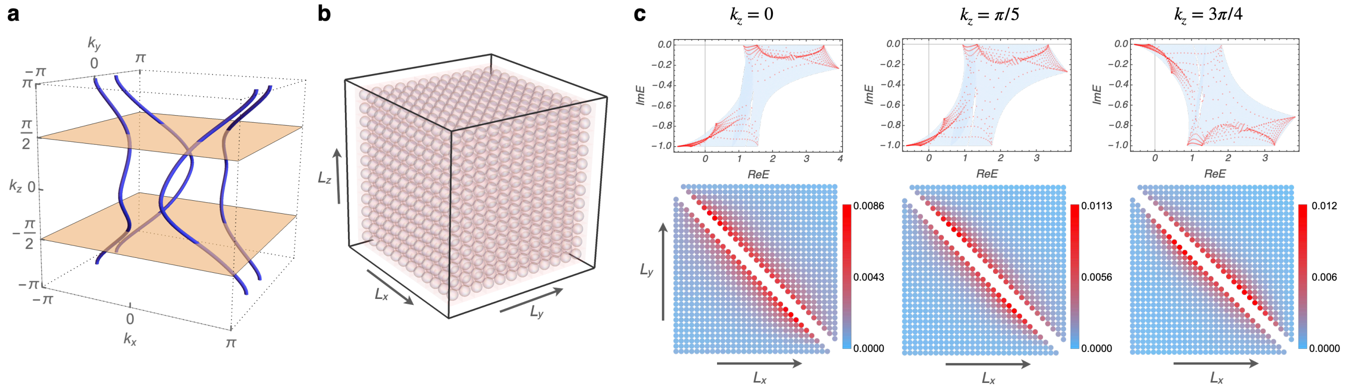

Supplementary Figure 12. The Hamiltonian Eq. (93) possesses four exceptional lines in 3D Brillouin zone shown in (a). The spatial distribution of eigenstates on the cube geometry ($L_x = L_y = L_z = 12$) is plotted in (b). For these two-dimensional subsystems with $k_z = 0, \pi/5, 3\pi/4$, we plot in (c) the periodic-boundary spectrum (the light blue region) and the open-boundary eigenvalues (the red dots) under the lower triangle geometry with system size $L_x = L_y = 30$. In addition, we show the spatial distribution of eigenstates on these triangle geometries.

Here, we take the non-Hermitian parameter γ as 1/4. We numerically calculate the spectra in Fig. 11(a)(c) and spatial distribution of the wave function in Fig. 11(b)(d) of the Hamiltonian Eq.(92) under different open-boundary geometries. It shows that the skin effect disappears under square geometry shown in Fig. 11(b), and reappears under diamond geometry in Fig. 11(d), which is the signature of GDSE. Note that the spectrum under square geometry (the red dots in Fig. 11(a)) coincides with the spectrum under periodic boundary condition (the gray dots in Fig. 11(a)(c)), while the spectrum under diamond geometry (the red dots in Fig. 11(a)) does not.

B. An example of GDSE in three dimensions

In this subsection, we construct a three-dimensional model with exceptional lines, and demonstrate that it exhibits the GDSE.

Here, we construct a three-dimensional model by coupling the two-dimensional photonic crystal model Eq.(92) along the z axis. In this model, we can observe four exceptional lines crossing the Brillouin zone along k_z direction, meanwhile, the system exhibits GDSE. The Hamiltonian of this model reads

$$\mathcal{H}_{3D}(\mathbf{k}) = \boldsymbol{d}(k_x, k_y) \cdot \boldsymbol{\sigma} - i\gamma/2(\sigma_0 - \sigma_z) + \cos k_z\, \sigma_z. \tag{93}$$

Here, we choose $\gamma = 1$, the only non-Hermitian parameter. The other parameters are the same as that in the model Eq.(92). In this 3D model, the coupling term along z direction is $\cos k_z\, \sigma_z$. The Hamiltonian possesses four exceptional lines in 3D Brillouin zone, which are plotted as the blue lines in Fig. 12(a). When $k_z = \pm\pi/2$, the Hamiltonian Eq. (93) reduces to two-dimensional photonic crystal model Eq.(92) with four exceptional points. Next we demonstrate that the system exhibits GDSE.

By definition, the sufficient and necessary condition for the existence of GDSE is that both of the following two points are satisfied: (i) the spectral area is nonzero; (ii) there is at least one geometry under which the skin effect disappear. The first point is satisfied due to the presence of the stable exceptional lines. The second point can be satisfied when we put the Hamiltonian on the cube geometry in Fig. 12(b), which is explained as follows.

First, the presence of the mirror symmetry, $\mathcal{H}(k_x, k_y, k_z) = \mathcal{H}(k_x, k_y, -k_z)$, forbids the skin effect along z axis of the cube geometry. Second, for each fixed k_z, $\cos k_z$ can be absorbed into μ_z, and the two-dimensional subsystem has no skin effect with square geometry. Therefore, with the cube geometry, the three-dimensional Hamiltonian does not exhibit skin effect, which is also verified by the numerical calculation. As shown in Fig. 12(b), the spatial distribution of eigenstates $W(\mathbf{x}) = \frac{1}{N}\sum_n |\psi_n(\mathbf{x})|^2$ is uniform on the cube geometry, with system size $L_x = L_y = L_z = 12$. The two points have been satisfied, therefore, the model has GDSE.

As a consequence of GDSE, the skin effect will reappear under other geometries. For example, if we cut the cube into two right-angle triangular prisms along z axis, the eigenstates will concentrate on the cross-section. Numerically, we calculate several two-dimensional subsystems with some generic k_z, and observe that skin modes will reappear under the triangular geometry, as shown in Fig. 12(c). It implies the appearance of the skin modes on the cross-sections of two triangular prisms.

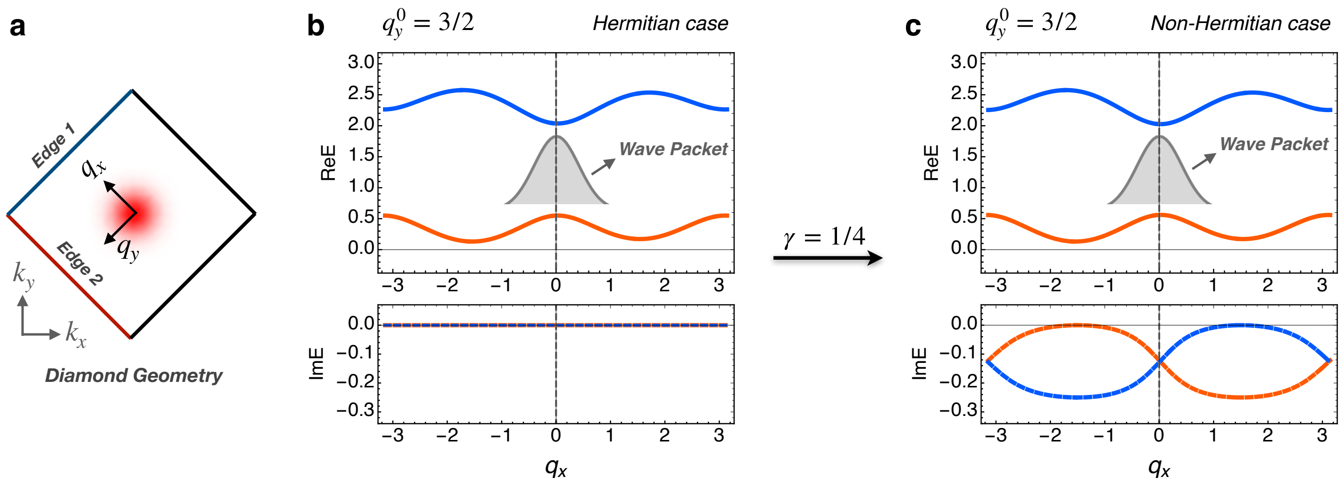

Supplementary Figure 13. (a) The Gaussian wave packet is centered at the diamond geometry at the initial time, and the momentum basis q_x and q_y are parallel to edge 2 and edge 1, respectively. The q_y^0-component of the Gaussian wave packet has a plane-wave form in q_y direction and Gaussian form in q_x direction illustrated as the gray wave packet centered at $q_x = 0$ in (b)(c). The real- and imaginary-part band structures of $\mathcal{H}(q_x, q_y^0)$ in Eq.(95) are shown in (b) when $\gamma = 0$, and are plotted in (c) when $\gamma = 1/4$.

C. Wave-packet dynamics

In this subsection, we explain the transverse motion of wave packet shown in Fig. 4(d) in the main text, and comment on the time evolution of system state in classical wave system with gain and/or loss and dissipative open quantum system.

1. The transverse motion of wave packet induced by skin effect

We state that the skin effect causes some components of the wave packet to drift parallel to the edge in red color in Fig. 13(a), and suppresses the reflection components perpendicular to this edge. Therefore, after several bounces between the edges, the wave packet finally appears as a transverse motion shown in Fig. 4(d) in the main text.

In our example, the wave packet has Gaussian form centered at $\mathbf{k}_c = (-2, -2)$ in momentum space, and evolves according to

$$|\psi_t\rangle = \mathcal{N}(t) e^{-i\mathcal{H}_{\rm OBC} t} |\psi_0\rangle. \tag{94}$$

Therefore, the wave packet will hit the edge in red color in Fig. 13(a) and scattering off it. In this process, the momentum component of the wave packet parallel to the edge is conserved. Hence, we transform the momentum basis from (k_x, k_y) to (q_x, q_y), as shown in Fig. 13(a), where $\mathbf{k} = S\mathbf{q}$ with $S = \{\{-1, -1\}, \{1, -1\}\}$. In $\mathbf{q}$ basis, the Gaussian wave packet is centered at $\mathbf{q}_c = (0, 2)$. Accordingly, the Hamiltonian Eq.(92) can be rewritten in $\mathbf{q}$ basis,

$$\tilde{\mathcal{H}}(q_x, q_y) = \mathcal{H}(S\,\mathbf{k}) = \boldsymbol{d}(-q_x - q_y, q_x - q_y) \cdot \boldsymbol{\sigma} - i\gamma/2(\sigma_0 - \sigma_z). \tag{95}$$

For a given q_y (here we select $q_y = q_y^0 = 3/2$), the spectral winding number $w_{E_b}(q_y^0)$ is nonzero, which implies the skin effect on the edge 1 in Fig. 13(a). Next, we will show that this skin effect results in the transverse drift of some components of the wave packet.

- The transverse drift of the wave packet from the skin effect on edge 1

  We focus on the component that has plane-wave form in q_y direction and Gaussian form in q_x direction. Note that the actual two-dimensional Gaussian wave packet is the coherent superposition of these components with a certain weight. Here we select the component with $q_y^0 = 3/2$. In q_x direction, the component is a Gaussian wave packet centered at $q_x = 0$ as shown in Fig. 13(b)(c). In Hermitian case ($\gamma = 0$), the component will disperse

with zero group velocity in q_x direction. In Fig. 4(c) of the main text, therefore, the Gaussian wave packet always slowly disperses with time in q_x direction.

However, in non-Hermitian case ($\gamma = 1/4$), the component will have a drift along the positive q_x direction. As shown in Fig. 13(c), around $q_x = 0$ each energy band has symmetric real part but asymmetric imaginary part, which corresponds to the presence of skin effect. In the range from $q_x = 0$ to 1, the energy band in blue color has a larger imaginary part and positive group velocity ($\partial \operatorname{Re} E/\partial q_x > 0$), while in the range from $q_x = -1$ to 0, the red-color energy band has a larger imaginary part and positive group velocity. The components with larger imaginary part decay more slowly and therefore dominate the evolution of the wave packet as time goes on. Consequently, some components of the Gaussian wave packet have a transverse drift along the positive q_x direction as shown in Fig. 4(d) of the main text, which is different from the Hermitian case.

Next, we will simply state that the suppression of the reflection wave components off the edge 2 ascribes to the skin effect on this edge.

- The suppression of the reflection wave components from the skin effect on edge 2

  In the process of wave packet hitting the boundary and scattering off, q_x is preserved. Therefore, we consider the wave-packet component that has plane-wave form in q_x direction and Gaussian form in q_y direction, labeled by q_x-component. The actual two-dimensional Gaussian wave packet is composed of these q_x-components with different weight. The skin effect on edge 2 means that for a given q_x, the spectral winding number is nonzero, which suppresses the reflection wave of the q_x-component.

To sum up, due to the skin effect on the edge 1 and edge 2, the Gaussian wave packet, going through several bounces between the two edges (edge 1 and the other parallel edge), finally appears as a transverse motion.

2. A comment on the time evolution in non-Hermitian system

In classical wave system, the evolution of a system state is governed by wave equation formally analogous to the Schrödinger equation [15]. In the cases with gain and/or loss, like the photonic crystal model in Ref. [16], the time evolution of a localized excitation (wave packet) is nonunitary, which can be captured by an effective non-Hermitian matrix [18]. Therefore, we believe that the phenomena in the simulation of wave-packet dynamics, e.g., the anomalous dynamical behavior shown in Fig. 4(d) of the main text, can be observed in a realistic classical wave system.

In the (driven or dissipative) open quantum system, a system state is described by the density matrix $\hat{\rho}$, whose time evolution is governed by the Lindblad master equation [19],

$$\frac{d\hat{\rho}}{dt} = -i[\hat{H}, \hat{\rho}] + \sum_x (2\hat{L}_x \hat{\rho} \hat{L}_x^\dagger - \{\hat{L}_x^\dagger \hat{L}_x, \hat{\rho}\}). \tag{96}$$

Here $\hat{H} = \sum_{xy} \mathcal{H}_{xy} \hat{c}_x^\dagger \hat{c}_y$ is the system Hamiltonian, and $\hat{L}_x = \sum_y \mathcal{D}_{xy} \hat{c}_y$ is the Lindblad dissipators describing quantum jumps due to coupling to the environment. In this setting, the dynamics of the density matrix $\hat{\rho}(t)$ can be capture by the single-particle correlation $C_{xy}(t) = \mathrm{tr}[\hat{\rho}(t)\hat{c}_x^\dagger \hat{c}_y]$. After some tedious calculations, one obtains that the time evolution of the correlation follows [20, 21]

$$C(t) = G(t)C(0)G^\dagger(t); \quad G(t) = e^{i\mathcal{H}_{\text{eff}} t}, \tag{97}$$

where $\mathcal{H}_{\text{eff}} = (\mathcal{H} - i\,\mathcal{D}^\dagger \mathcal{D})^t$ is the effective non-Hermitian matrix, the superscript "t" representing the transpose of the matrix. In the closed (Hermitian) quantum system, the system has no coupling with the external environment, and the density matrix evolves according to $d\hat{\rho}(t)/dt = -i[\hat{H}, \hat{\rho}]$. As a result, the dynamics of correlation satisfies

$$C(t) = G(t)C(0)G^\dagger(t); \quad G(t) = e^{i\mathcal{H}^t t}, \tag{98}$$

where $\mathcal{H}^t$ is transpose of the Hamiltonian matrix $\mathcal{H}$. Comparing Eq.(97) with Eq.(98), we conclude that the time evolution of the correlation $C(t)$ in dissipative open quantum system has the same form as the Hermitian case.

---

[1] Loomis, L. H. & Sternberg, S. *Advanced calculus* (World Scientific, 1968).

[2] Zhang, K., Yang, Z. & Fang, C. Correspondence between Winding Numbers and Skin Modes in Non-Hermitian Systems. *Phys. Rev. Lett.* **125**, 126402 (2020).
[3] Okuma, N., Kawabata, K., Shiozaki, K. & Sato, M. Topological Origin of Non-Hermitian Skin Effects. *Phys. Rev. Lett.* **124**, 086801 (2020).
[4] Kawabata, K., Shiozaki, K., Ueda, M. & Sato, M. Symmetry and Topology in Non-Hermitian Physics. *Phys. Rev. X* **9**, 041015 (2019).
[5] Kawabata, K., Okuma, N. & Sato, M. Non-Bloch band theory of non-Hermitian Hamiltonians in the symplectic class. *Phys. Rev. B* **101**, 195147 (2020).
[6] Hofmann, T. *et al.* Reciprocal skin effect and its realization in a topolectrical circuit. *Phys. Rev. Research* **2**, 023265 (2020).
[7] Lee, C. H., Li, L. & Gong, J. Hybrid Higher-Order Skin-Topological Modes in Nonreciprocal Systems. *Phys. Rev. Lett.* **123**, 016805 (2019).
[8] Kawabata, K., Sato, M. & Shiozaki, K. Higher-order non-Hermitian skin effect. *Phys. Rev. B* **102**, 205118 (2020).
[9] Yao, S. & Wang, Z. Edge States and Topological Invariants of Non-Hermitian Systems. *Phys. Rev. Lett.* **121**, 086803 (2018).
[10] Yokomizo, K. & Murakami, S. Non-Bloch Band Theory of Non-Hermitian Systems. *Phys. Rev. Lett.* **123**, 066404 (2019).
[11] Yang, Z., Zhang, K., Fang, C. & Hu, J. Non-Hermitian Bulk-Boundary Correspondence and Auxiliary Generalized Brillouin Zone Theory. *Phys. Rev. Lett.* **125**, 226402 (2020).
[12] Yang, Z., Chiu, C.-K., Fang, C. & Hu, J. Jones Polynomial and Knot Transitions in Hermitian and non-Hermitian Topological Semimetals. *Phys. Rev. Lett.* **124**, 186402 (2020).
[13] Yang, Z., Schnyder, A. P., Hu, J. & Chiu, C.-K. Fermion Doubling Theorems in Two-Dimensional Non-Hermitian Systems for Fermi Points and Exceptional Points. *Phys. Rev. Lett.* **126**, 086401 (2021).
[14] Shen, H., Zhen, B. & Fu, L. Topological Band Theory for Non-Hermitian Hamiltonians. *Phys. Rev. Lett.* **120**, 146402 (2018).
[15] Özdemir, Ş. K., Rotter, S., Nori, F. & Yang, L. Parity–time symmetry and exceptional points in photonics. *Nature Materials* **18**, 783–798 (2019).
[16] Zhou, H. *et al.* Observation of bulk Fermi arc and polarization half charge from paired exceptional points. *Science* **359**, 1009–1012 (2018).
[17] Denner, M. M. *et al.* Exceptional topological insulators. *Nature Communications* **12**, 5681 (2021).
[18] Ashida, Y., Gong, Z. & Ueda, M. Non-hermitian physics. *Advances in Physics* **69**, 249–435 (2020).
[19] Dalibard, J., Castin, Y. & Mølmer, K. Wave-function approach to dissipative processes in quantum optics. *Phys. Rev. Lett.* **68**, 580–583 (1992).
[20] Song, F., Yao, S. & Wang, Z. Non-Hermitian Skin Effect and Chiral Damping in Open Quantum Systems. *Phys. Rev. Lett.* **123**, 170401 (2019).
[21] Mao, L., Deng, T. & Zhang, P. Boundary condition independence of non-Hermitian Hamiltonian dynamics. *Phys. Rev. B* **104**, 125435 (2021).
